# Integrative Analysis Extracts a Core ceRNA Network of the Fetal Hippocampus With Down Syndrome

**DOI:** 10.3389/fgene.2020.565955

**Published:** 2020-11-30

**Authors:** Shengran Wang, Xia Tang, Litao Qin, Weili Shi, Shasha Bian, Zhaokun Wang, Qingqing Wang, Xin Wang, Jianqin Gu, Bingtao Hao, Keyue Ding, Shixiu Liao

**Affiliations:** ^1^Medical Genetic Institute of Henan Province, Henan Provincial Key Laboratory of Genetic Diseases and Functional Genomics, National Health Commission Key Laboratory of Birth Defects Prevention, People’s Hospital of Zhengzhou University, Zhengzhou, China; ^2^Henan Provincial People’s Hospital, School of Medicine, Henan University, Zhengzhou, China; ^3^Henan Key Laboratory of Genetic Diseases and Functional Genomics, National Health Commission Key Laboratory of Birth Defects Prevention, Henan Provincial People’s Hospital, Zhengzhou, China; ^4^School of Medicine, Henan University, Zhengzhou, China

**Keywords:** Down syndrome (DS), hippocampus, competing endogenous RNAs (ceRNAs), single-cell RNA sequencing, epigenetic

## Abstract

Accumulating evidence suggests that circular RNAs (circRNAs)—miRNA–mRNA ceRNA regulatory network—may play an important role in neurological disorders, such as Alzheimer’s disease (AD). Interestingly, neuropathological changes that closely resemble AD have been found in nearly all Down syndrome (DS) cases > 35 years. However, few studies have reported circRNA transcriptional profiling in DS cases, which is caused by a chromosomal aberration of trisomy 21. Here, we characterized the expression profiles of circRNAs in the fetal hippocampus of DS patients (*n* = 8) and controls (*n* = 6) by using microarray. MiRNA, mRNA expression profiling of DS from our previous study and scRNA-seq data describing normal fetal hippocampus development (GEO) were also integrated into the analysis. The similarity between circRNAs/genes with traits/cell-types was calculated by weighted correlation network analysis (WGCNA). miRanda and miRWalk2 were used to predict ceRNA network interactions. We identified a total of 7,078 significantly differentially expressed (DE) circRNAs, including 2,637 upregulated and 4,441 downregulated genes, respectively. WGCNA obtained 15 hub circRNAs and 6 modules with cell type–specific expression patterns among scRNA-seq data. Finally, a core ceRNA network was constructed by 14 hub circRNAs, 17 DE miRNA targets and 245 DE mRNA targets with a cell type–specific expression pattern annotation. Known functional molecules in DS or neurodegeneration (e.g., miR-138, OLIG1, and TPM2) were also included in this network. Our findings are the first to delineate the landscape of circRNAs in DS and the first to effectively integrate ceRNA regulation with scRNA-seq data. These data may provide a valuable resource for further research on the molecular mechanisms or therapeutic targets underlying DS neuropathy.

## Introduction

Down syndrome (DS) is one of the leading causes of congenital intellectual disability and cognitive impairment due to a naturally occurring extra copy of chromosome 21, i.e., trisomy 21 (Zhao Y. et al., 2017). DS occurs in approximately 1 in 700 live births, and its incidence increases with a high maternal age (Carothers et al., 1999). DS involves a remarkably broad spectrum of human disorders (Capone et al., 2016; Zhao Y. et al., 2017), including chronic systemic inflammation, lupus erythematosus, amyotrophic lateral sclerosis, various cancers, and autism spectrum disorder (Zis et al., 2017). The neuropathological changes noted in nearly all DS cases > 35 years closely resemble Alzheimer’s disease (AD) (Tramutola et al., 2020), suggesting an overlap of the underlying molecular mechanisms between these two disorders. Understanding DS’s pathogenesis could ultimately provide novel therapeutic targets for this condition (Yu et al., 2018).

The hippocampus is a vital organ involved in memory formation and consolidation. Pathological changes of the neurogenesis impairment, such as hypocellularity in the hippocampus, were significant causes of the functional alteration observed in DS patients (Guidi et al., 2008). The epigenetic changes mediated by miRNAs in the hippocampus have been demonstrated in DS, e.g., miR-155 targeting *CFH* may protect neurons from axonal injury (Brás et al., 2018). Our previous work found that the upregulated miR-138 in the hippocampal tissues of fetal DS results in a lower expression of *EZH2* (Shi et al., 2016). Recently, more attention has been paid to a new class of noncoding RNA [i.e., circular RNAs (circRNAs) (Li et al., 2018)] with multiple molecular functions, such as competing with linear RNAs in the splicing, deriving pseudogenes, working as endogenous miRNA sponges (Jiang et al., 2019), and encoding proteins (Yang et al., 2018; Wang et al., 2019). CircRNAs are expressed in a tissue-specific manner and appear to be specifically enriched in the central nervous system. During neuronal development, the expression of circRNAs is regulated by synaptic plasticity, highlighting its specific neuronal functions (Chen and Schuman, 2016). For example, ciRS-7 may act as an miRNA-7 sponge that is involved in a significantly dysregulated circuit of ciRS-7-miRNA-7-*UBE2A* in AD (Zhao et al., 2016).

To the best of our knowledge, the expression profiles and potential function of circRNAs in the fetal hippocampus from DS patients remain undefined. Accordingly, we characterized the different expression profiles of circRNAs in the fetal hippocampus with DS using the microarray analysis to delineate the ceRNA network in DS. We then analyzed scRNA-seq data of normal fetal hippocampus development (GSE131258) to study the cell clustering. The circRNA expression profiles and scRNA-seq data were further used to construct coexpression networks, and 15 hub circRNAs and six modules with cell type–specific expression patterns were obtained, respectively. Overlap of genes in these six modules and DE mRNAs in DS were filtered for further analysis. We finally identified 14 hub circRNAs, 17 DE miRNA targets, and 245 DE mRNA targets to construct a core ceRNA network with cell type–specific expression pattern annotation. Our findings may provide clues for future research on novel mechanisms and therapeutic targets of DS nervous pathogenesis.

## Materials and Methods

### Clinical Specimens and Data

The fetal brain was obtained at the abortion of eight participants at 17–23 weeks of pregnancy that were diagnosed with trisomy 21 by amniocentesis (i.e., karyotype analysis and chromosomal aneuploidy test). We also obtained the fetal brain of six diploid embryos without any chromosomal abnormalities due to an accidental spontaneous abortion at the pregnancy of 17–23 weeks. The hippocampus was dissected and frozen in liquid nitrogen. All tissue donors provided their written informed consent, and the study was approved by the Human Ethics Committee of the People’s Hospital of Zhengzhou University.

Single-cell RNA (scRNA) sequencing data from human fetal hippocampus was available from the Gene Expression Omnibus (GEO)^1^ under the accession number GSE131258.

We used the ceRNAs and scRNA sequencing data to construct a core ceRNA network with cell type–specific expression pattern annotation. The flow chart of the study design is shown in Supplementary Figure S1.

### RNA Extraction, Library Preparation, and circRNA Microarray Hybridization

Total RNA of all 14 fetal hippocampus tissues (i.e., eight DS patients and six controls) were isolated and purified using Trizol reagent (Invitrogen, Carlsbad, CA, United States) and the NucleoSpin RNA clean-up Kit (MACHEREY-NAGEL, Duren, Germany). RNA disposal and library preparation was processed as previously described (Zhao Z. et al., 2017). The total amount and purity of the RNA were determined by a spectrophotometer (NanoDrop ND-2000, Thermo Fish Scientific, Waltham MA, United States). The RNA integrity was determined by 1% formaldehyde denaturing gel electrophoresis (RNA 6000 Nano Lab-on-a-Chip kit). After quality control, RNA was reverse-transcribed into cDNA using random primers containing the T7 promoter (First-Strand Enzyme Mix Kit). Then, the DNA–RNA mixture was transformed into the second strand DNA by the Second Strand Enzyme Mix. The DNA was used as a template to obtain cRNA by the T7 enzyme mix. The quality-controlled cRNA was used as a template to synthesize cDNA fluorescently labeled by Klenow fragment enzyme combined with random primers and dNTP with fluorescent tags (Cy3-dCTP). After purification, the labeled DNAs were hybridized onto a human circRNA array (CapitalBio Technology, v2.0) (Ouyang et al., 2017), containing approximately 175,807 human circRNAs.

### Analysis of Differentially Expressed circRNAs

We used Feature Extraction (v12, Agilent Technology) to extract expression signals for the microarray (Tan et al., 2014), and selected the circRNAs present at each sample (detected by FEFlag) for further analyses. GeneSpring GX (Agilent) was used for DE analysis. Significantly DE circRNAs were defined as the absolute of logarithm of fold change (log2| FC|) > 1.0 and adjusted *p* < 0.05. We used the WebGestalt webserver (Wang et al., 2017) to perform over-representation enrichment analysis based on databases of Gene Ontology (GO), pathway (KEGG), and disease (DisGeNET and OMIM) to investigate the potential function of DE circRNAs by inputting their parent genes and human genome 19 (hg19) reference. The circRNA-sponged miRNAs were predicted using miRanda (Witkos et al., 2011) and starBase^2^. We predicted *in silico* mRNAs potentially regulated by the DE circRNAs (through sponging miRNA) using miRWalk2 (Hang et al., 2019), by combining DE miRNA and mRNA in a subset of the samples in our previous study (Shi et al., 2016).

### Identification of Cell Type Using scRNA Data

We filtered scRNA sequencing data using Seurat (v3.1.2) in *R*. Only cells that expressed more than 800 genes and fewer than 7,000 genes were considered, and only genes expressed in at least 30 single cells were included for further analysis. Cells with a percentage of mitochondrial genes to total genes less than 15% were included. The unique molecular identifier (UMI) counts for each gene were normalized by default (i.e., NormalizeData function in Seurat). We also performed the scaling, linear dimensionality reduction, clustering, and visualization using ScaleData, JackStrawPlot, FindClusters, and RunUMAP in Seurat (v3.1.2). Due to computational intensity, we only filtered the top 40 cells per cluster (cells were ordered by barcode to guarantee the randomization of filter) in each sample, respectively. In total, 2,054 hippocampal cells remained for subsequent analysis. Cell clustering of these 2,054 single cells was identified using the same algorithm.

### Weighted Gene Co-expression Network Analysis (WGCNA)

Both circRNAs and scRNA sequencing data were analyzed by WGCNA, implemented in *R* (v1.68). Because network analysis is computationally intensive, we used 7,078 DE circRNAs and the top 3,000 highly variable genes obtained from scRNA data before network construction. The Topological Overlap Matrix (TOM) was created to describe the network interconnectivity or coexpression between each pair of features in relation to all others in the network. The parameters of deepsplit and minimum module size were set to 0 and 30, respectively. Only modules with a highly significant correlation between gene significance (GS) and module membership (MM) (cor > 0.4, *p* < 0.05) were filtered to further analysis. Nodes with the top 5 MM values were highlighted in the network.

### circRNA, miRNA, and mRNA Regulation Network Construction

For circRNAs, we investigated the potential function by their parent genes and predicted miRNAs. Based on the reciprocal interaction between our DE circRNAs and their predicted nine miRNA targets, combined with DE miRNAs identified in a subset of samples described in our DS cohort previously, we established a circRNA–miRNA network using Cytoscape v3.7.0 (Hang et al., 2019). The post-transcription regulation between miRNA and mRNA was endogenously competed by circRNA. We also constructed a circRNA–miRNA–mRNA interaction network, according to the most significantly predicted bilateral interactions integrated with WGCNA results between them. The DE features mRNAs identified in the subset sample were also included. All DE features (including circRNAs, miRNAs, and mRNAs) were highlighted in the network.

### Validation of Target Genes *in silico*

Target genes in the core ceRNA network were validated in an AD database (AlzData^3^) (Xu et al., 2018).

### Experimental Validation for Selected circRNAs

We selected five significant DE circRNAs and three circ-GRIK1s for experimental expression validation using qRT-PCR, with three additional hippocampus tissues from DS patients. We used SYBR Green assays in a total reaction volume of 10 μl for 40 cycles. β-actin was used as an inner reference. The relative expression level of each circRNA was calculated using 2^–Δ^ ^Δ^ ^*Ct*^, followed by Student’s *t*-tests for significantly differential expression calculation (Yang et al., 2020). The primers are listed in Supplementary Table S1.

### Dual-Luciferase Reporter Assay

The hsa_circ_0078328 fragments covering wild-type and mutant miR-138-5p binding sites were inserted into the basic vector psiCHECK−2 vector (Hanbio Biotechnology), forming the wild-type (hsa_circ_0078328-WT) and mutant-type (hsa_circ_0078328-MUT) luciferase reporter plasmids. Then, the generated reporter plasmids were cotransfected with miR-138-5p mimics or miR−NC into 293T cells, respectively. The medium was changed after 6 h. The luciferase activity was analyzed via a dual-luciferase analysis system (Promega) after 48 h, according to the manufacturer’s instructions. Three independent transfection experiments were performed.

## Results

### Microarray Identified Significantly Differentially Expressed circRNAs

**FIGURE 1:**
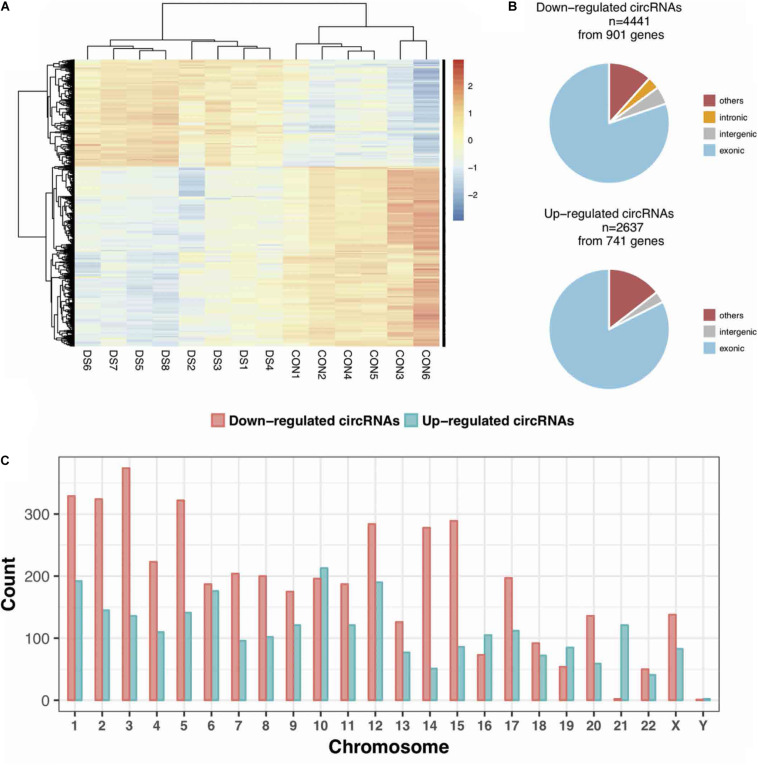
The expression profiles of circRNAs in the fetal hippocampus of DS patients and controls. **(A)** A heatmap showing the hierarchical clustering of differentially expressed (DE) circRNAs between two groups. **(B)** The categories of DE circRNAs according to its annotation. **(C)** The distribution of DE circRNAs on chromosomes.

The expression profiles of circRNAs were characterized in the fetal hippocampus of eight DS patients and six diploid embryos. After normalization, the distribution of the overall expression levels of circRNAs was similar between the DS patients and diploid embryos, and DE circRNAs were identified (Supplementary Figures S2A–C). Of a total of 175,807 expressed circRNAs, 7,078 significant DE circRNAs were identified for further analyses, including 2,637 upregulated and 4,441 downregulated, respectively (Figure 1A). Significant DE circRNAs (adjusted *p* < 0.01 and log2| FC| > 1) are listed in Supplementary Table S2. The DE circRNAs were classified into four types according to their sequence location (i.e., exon, introns, intergenic, and others), suggesting that the majority of DE circRNAs were transcribed from exonic regions (Figure 1B). The distribution of circRNAs along the chromosomes is summarized in Figure 1C. Most of the DE circRNAs were located on chromosome 21 with upregulation, consistent with the trisomy 21. We list the 20 most significant DE circRNAs (according to log2| FC|) in Table 1, containing multiple circRNAs generated from the same parental gene. For example, eight upregulated circRNAs were derived from the tumor suppressor *GPC5*. We also noted that *GPR98*—a gene related to the Usher syndrome of DS phenotype (Zhao Y. et al., 2017; Wei et al., 2018)— generated 13 downregulated circRNAs.

**TABLE 1:** Top 20 upregulated and downregulated circRNAs.

Probe	Chr	Symbol	Adjusted *p*	Log2FC
hsa-circRNA2166-1	13	GPC5	2.24E-02	3.884989
hsa-circRNA2166-8	13	GPC5	3.01E-02	3.680999
hsa_circ_0100987	13	GPC5	1.73E-02	3.475474
hsa-circRNA2166-3	13	GPC5	2.37E-02	3.379621
hsa-circRNA2166-7	13	GPC5	3.44E-02	3.261531
hsa_circ_0100989	13	GPC5	1.90E-02	3.228973
hsa-circRNA2166-5	13	GPC5	2.65E-02	3.199123
hsa_circ_0100988	13	GPC5	3.94E-02	3.116365
hsa_circ_0136188	8	NEFL	2.49E-02	3.801573
hsa_circ_0136190	8	NEFL	1.25E-02	3.457988
hsa_circ_0136191	8	NEFL	1.60E-02	3.293959
hsa_circ_0136187	8	NEFL	1.79E-02	3.091531
hsa_circ_0043887	17	CNTNAP1	1.28E-02	3.575675
hsa_circ_0058363	2	TUBA4A	4.42E-02	3.430687
hsa_circ_0080947	7	SEMA3D	2.92E-02	3.252779
hsa-circRNA3152-8	16	CPNE7	1.21E-02	3.232047
hsa_circ_0131237	6	LPA	8.13E-03	3.149747
hsa_circ_0042551	17	ALDOC	3.52E-02	3.112533
hsa_circ_0131986	6	BEND6	1.80E-02	3.087802
hsa_circ_0095633	11	LUZP2	1.12E-02	3.069187
hsa_circ_0073309	5	GPR98	2.59E-02	−3.75617
hsa_circ_0073310	5	GPR98	3.18E-02	−3.62152
hsa_circ_0073325	5	GPR98	1.88E-02	−3.51677
hsa_circ_0073306	5	GPR98	3.39E-02	−3.48852
hsa_circ_0073290	5	GPR98	3.54E-02	−3.47832
hsa_circ_0073289	5	GPR98	2.68E-02	−3.46597
hsa_circ_0073304	5	GPR98	2.97E-02	−3.42304
hsa_circ_0129959	5	GPR98	2.78E-02	−3.36695
hsa_circ_0129954	5	GPR98	2.56E-02	−3.36345
hsa_circ_0073326	5	GPR98	2.45E-02	−3.36036
hsa_circ_0129974	5	GPR98	2.40E-02	−3.34809
hsa_circ_0073284	5	GPR98	3.77E-02	−3.32294
hsa_circ_0073291	5	GPR98	3.23E-02	−3.31658
hsa_circ_0135183	7	DLX6-AS1	4.97E-02	−3.72999
hsa_circ_0043539	17	TOP2A	1.36E-02	−3.52281
hsa-circRNA11458-30	17	TOP2A	1.40E-02	−3.46963
hsa_circ_0015764	1	ASPM	1.29E-02	−3.39534
hsa_circ_0015766	1	ASPM	1.63E-02	−3.35924
hsa_circ_0135184	7	DLX6-AS1	4.03E-02	−3.37768
hsa-circRNA11595-11	17	BRIP1	2.46E-02	−3.33256

### The Potential Function of DE circRNAs Illustrated by Parental Genes

**FIGURE 2:**
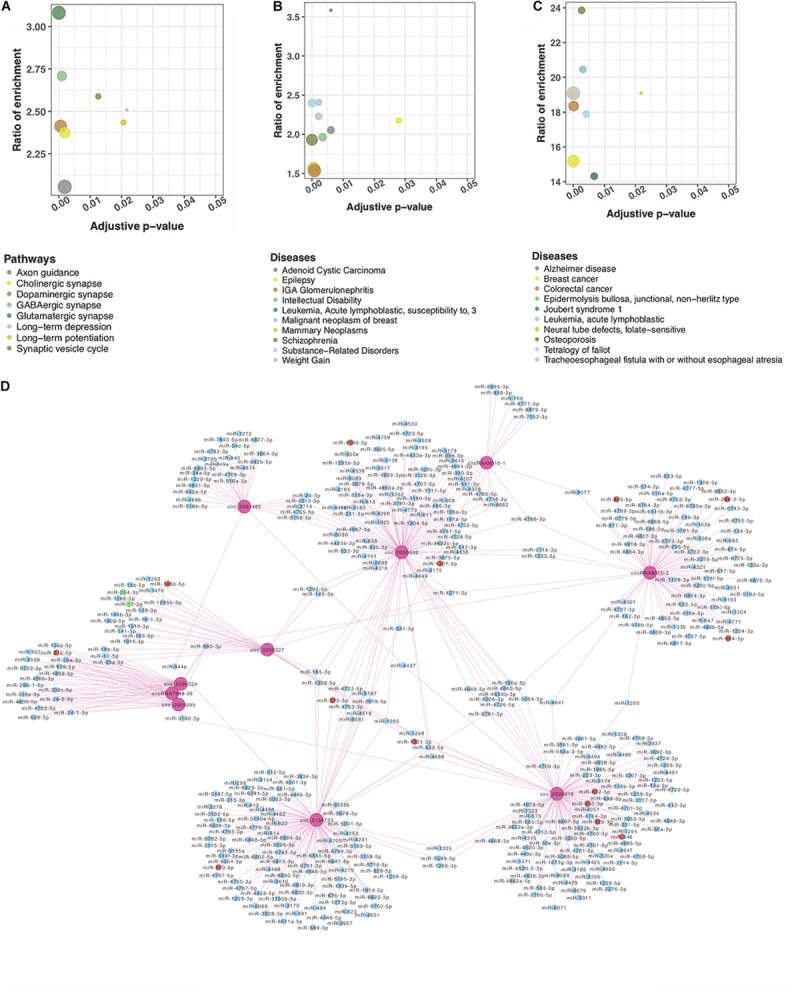
Functional network analysis of DE circRNAs according to their parent genes. Genes were significantly enriched in nervous system–associated terms that were identified by **(A)** KEGG; **(B)** DisGeNET, and **(C)** OMIM. **(D)** A network of circRNA–miRNA interaction of the top 10 DE circRNAs. Ten pink rounds represent circRNAs. The blue ones are their potential miRNA targets with no difference expression, red rounds represent upregulated potential targets, and the green ones are potentially downregulated targets.

circRNAs may regulate their parental genes’ transcription or post-transcription (Dang et al., 2017). We performed pathway and disease enrichment analysis for these genes using the databases of KEGG, DisGeNET, and OMIM. Based on KEGG, the parental genes of DE circRNAs were significantly enriched in nine pathways associated with the nervous system (adjusted *p* < 0.05) (Figure 2A). Of note, multiple pathways were related to synapse, e.g., glutamatergic, GABAergic, and synaptic vesicle cycle. Other functional pathways included axon guidance, retrograde endocannabinoid signaling, long-term potentiation (LTP), and long-term depression (LTD) (Figure 2A). All these pathways suggest a potential role of circRNAs in the neural signaling transfer underlying the DS pathogenesis (Kleschevnikov et al., 2004; Scott-McKean and Costa, 2011; Lysenko et al., 2018; Moretto et al., 2018). The analysis on DisGeNET showed that “intellectual disability” is significantly enriched related to the mental retardation of the DS patients (Figure 2B). “Alzheimer disease” obtained from the OMIM-based enrichment analysis also highlighted a potential role in the early onset of AD in the DS patients (Figure 2C). Our analysis indicates that DS is associated with a number of neurobiological pathways that may result in learning and memory deficits and early onset AD (Ruparelia et al., 2013).

### circRNAs Interacted With the Known DS Neurological Disorder–Related miRNAs

circRNAs may contain multiple miRNA binding sites capable of sequestering miRNAs with their own miRNA response elements, thereby acting as miRNA sponges to regulate targeted gene expression at the transcriptional or post-transcriptional levels (Hansen et al., 2013; Barrett and Salzman, 2016). The interaction of the top 10 significant DE circRNAs (based on adjusted *p*) with the predicted miRNAs (*n* = 504) suggests that a given circRNA may have multiple targeted miRNAs (Figure 2D). Four previously reported DS-related miRNAs [i.e., miR-155-5p (Lu et al., 2013), miR-146a-5p (Arena et al., 2017), miR-138-5p (Shi et al., 2016), and miR-802 (Brás et al., 2018)], were predicted to obviously interact with DE circRNAs. The top 20 significant DE circRNAs for each miRNA are shown in Table 2.

**TABLE 2:** Known miRNAs associated with DS and their interacted circRNAs.

miRNA	circRNA		
hsa-miR-155-5p	hsa_circ_0067470	hsa_circ_0035485	hsa_circ_0035488
	hsa_circ_0023610	hsa_circ_0035487	hsa_circ_0073445
	hsa_circ_0118623	hsa_circ_0067469	hsa_circ_0073443
	hsa_circ_0054554	hsa_circ_0120335	hsa_circ_0067472
	hsa_circ_0118117	hsa_circ_0138451	hsa_circ_0122061
	hsa_circ_0070210	hsa_circ_0024319	hsa_circ_0100932
	hsa_circ_0118118	hsa_circ_0100931	
hsa-miR-138-5p	hsa_circ_0136728	hsa_circ_0078328	hsa_circ_0035653
	hsa_circ_0131090	hsa-circRNA7492-17	hsa-circRNA8910-13
	hsa_circ_0091053	hsa_circ_0115879	hsa_circ_0140138
	hsa_circ_0022404	hsa_circ_0035649	hsa_circ_0040304
	hsa_circ_0104117	hsa_circ_0002138	hsa_circ_0074859
	hsa_circ_0109005	hsa_circ_0110582	hsa_circ_0138111
	hsa_circ_0055868	hsa_circ_0031781	
hsa-miR-146a-5p	hsa-circRNA12419-2	hsa_circ_0020955	hsa_circ_0133379
	hsa-circRNA14521	hsa_circ_0022092	hsa_circ_0104843
	hsa-circRNA1203-20	hsa_circ_0020956	hsa_circ_0128323
	hsa_circ_0052850	hsa_circ_0015762	hsa_circ_0129907
	hsa-circRNA2719-14	hsa_circ_0005383	hsa_circ_0039257
	hsa_circ_0085586	hsa_circ_0015760	hsa_circ_0085389
	hsa_circ_0005365	hsa_circ_0071467	
hsa-miR-802	hsa_circ_0056745	hsa-circRNA12735-2	hsa-circRNA1203-7
	hsa_circ_0127798	hsa-circRNA1203-24	hsa-circRNA5535-9
	hsa_circ_0027885	hsa-circRNA6265-4	hsa_circ_0005439
	hsa_circ_0065029	hsa-circRNA6411-18	hsa_circ_0006084
	hsa_circ_0065031	hsa_circ_0095820	hsa-circRNA3109-14
	hsa_circ_0097862	hsa_circ_0024603	hsa-circRNA5535-11
	hsa_circ_0065030	hsa-circRNA13641-17	

**FIGURE 3:**
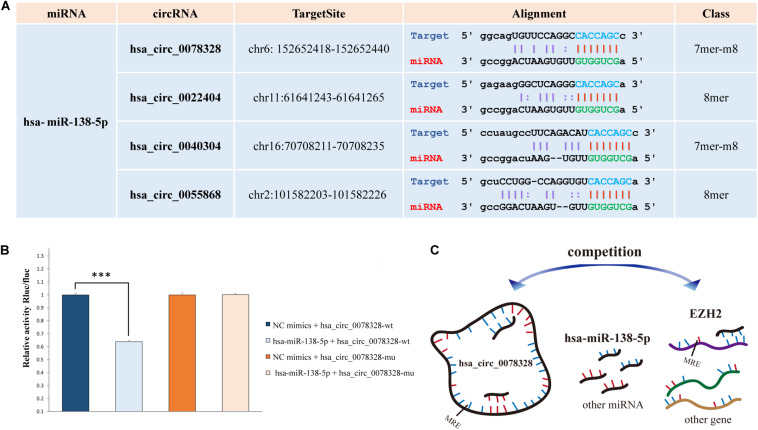
Interactions of *hsa-miR-138-5p*. **(A)** The predicted binding site of *hsa-miR-138-5p* with the circRNAs list in Table 2. **(B)** The interaction between *hsa_circ_0078328* and *hsa-miR-138-5p* was evaluated by the dual-luciferase reporter assay. **(C)** The pattern of *hsa_circ_0078328/miR-138-5p/EZH2* interactions.

The *hsa-miR-138-5p* and its target *EZH2* may be involved in hippocampus neuropathy in DS patients as we reported previously (Shi et al., 2016). Thus, we predicted the binding site of the top 20 significant DE target circRNAs for hsa-miR-138-5p using starBase (four binding sites) (Figure 3A). The circRNA hsa_circ_0078328 was used to test whether it was a direct target of hsa-miR-138-5p. The luciferase assay showed that its relative activity was significantly decreased in 293T cells cotransfected with hsa_circ_0078328-WT and miR-138-5p mimics compared with the miR−NC group. However, there was no significant change in the luciferase activity in 293T cells cotransfected with hsa_circ_0078328-MUT and miR-182 mimics (Figure 3B). These results indicated that hsa_circ_0078328 was a direct target of hsa-miR-138-5p, and it served as a competing endogenous RNA to sponge miR-182 and finally participated in the regulation of *EZH2* expression (Figure 3C).

### Three circ-GRIK1s Located on Chromosome 21 May be Involved in the Abnormal Neurodevelopment in DS

**FIGURE 4:**
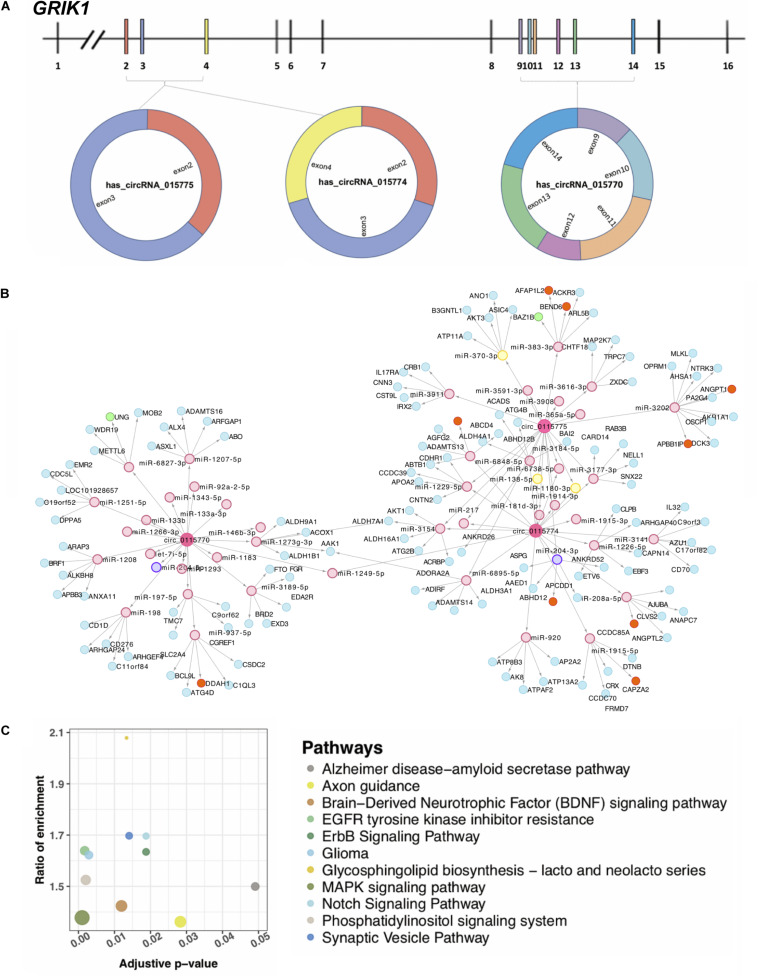
Three circRNAs (*hsa_circRNA_0115770, hsa_circRNA_0115774, and hsa_circRNA_0115775*) transcribed from *GRIK1*. **(A)** The gene structure of *GRIK1* and the three circRNAs transcribe in different manners. **(B)** A multidimensional network of circRNA–miRNA–mRNA. Three large rounds in pink represent circRNAs, small rounds in pink represent potential miRNA targets with no difference expression, yellow rounds represent potentially upregulated targets, and the purple is the potential downregulated target. The blue rounds represent their potential mRNA target with no difference in expression, orange rounds represent upregulated potential targets, and the green are downregulated potential targets. **(C)** Pathways are associated with the nervous system. The adjusted *p* represents the significance of the pathway.

*Grik1* triplication is a newly defined factor in causing cognitive impairment in DS (Valbuena et al., 2019). Three upregulated circRNAs (hsa_circRNA_0115770, hsa_circRNA_0115774, and hsa_circRNA_0115775) were transcribed from *GRIK1* on chromosome 21 (Figure 4A). Their different sequence constitutions suggest that they may have a portion of other targets supported by the network analysis (Figure 4B). For each circRNA, the top 20 targeted miRNAs were selected, and 47 unique miRNAs remained because at least two circRNAs targeted a given miRNA. The top five significant mRNAs predicted to be targeted by each circRNA-sponged miRNA based on 3′ UTR binding sites were collected. A total of 128 mRNAs targeted by 47 miRNAs were targeted by these three circRNAs. Of note, the majority of interactions in this network were unique. Through the proposed multidimensional network, circRNA may exert its cascade effect by regulating miRNAs and then mRNAs. Pathways associated with nervous pathogenesis are shown in Figure 4C.

### Validation of Microarray Expression Using qRT-PCR

**FIGURE 5:**
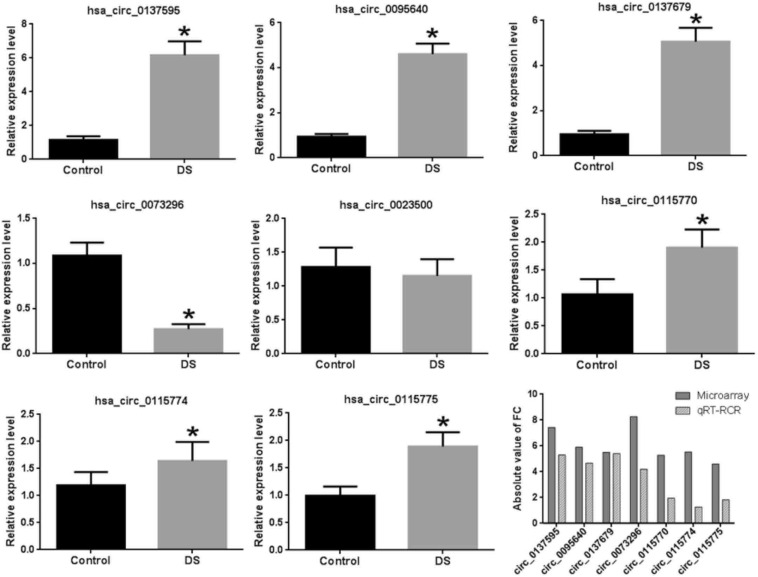
Validation of eight DE circRNAs by qRT-PCR. The asterisks indicate the statistically significant difference.

In addition to the three circ-GRIK1s, we also validated another five significant DE circRNAs, which were the first reported to be differentially expressed in neurological disease. Supplementary Figure S3 shows the circRNA–miRNA interaction of these five DE circRNAs. Our results demonstrated that seven of the eight significantly DE circRNAs were validated with similar expression results except for hsa_circ_0023500 (Figure 5); however, a larger cohort is needed for further validation.

### WGCNA Identified the Clustered circRNA Modules

**FIGURE 6:**
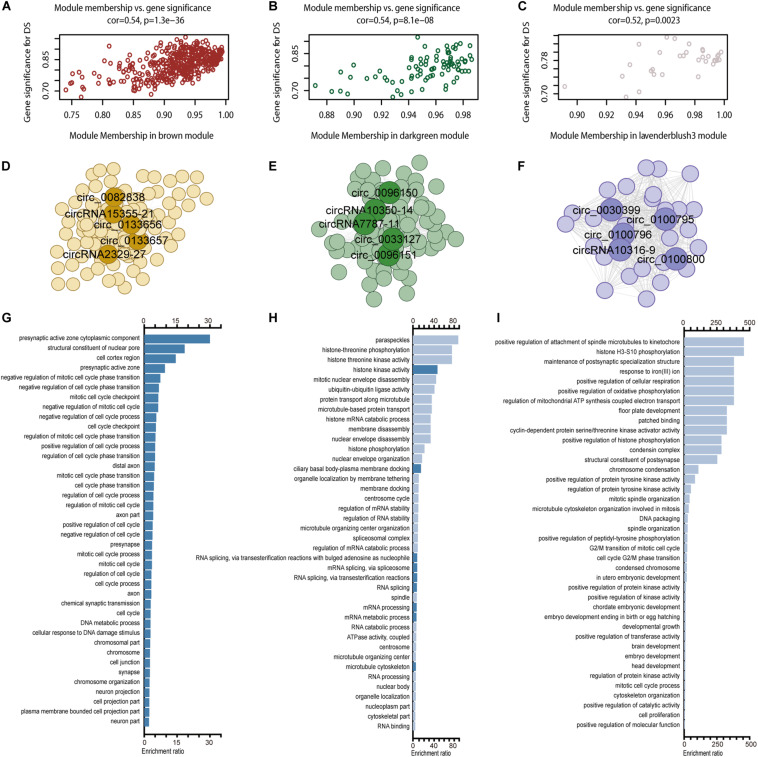
WGCNA for circRNAs. Including module membership versus gene significance correlation in the brown **(A)**, darkgreen **(B)**, and lavender blush 3 **(C)** modules; a coexpression network of the brown **(D)**, darkgreen **(E)**, and lavender blush 3 **(F)** module with five hub nodes’ circRNA ID marking in each module and GO enrichment results in brown **(G)**, darkgreen **(H)**, and lavender blush 3 **(I)** module.

circRNA modules were identified among DE circRNAs using WGCNA (Supplementary Figure S4A). The eigengene dendrogram is shown in Supplementary Figure S4B. GO analysis identified modules with critical biological functions, for example, circRNAs in brown, darkgreen, lavender blush 3, pink, black, and green modules were associated with the biological regulation of the nervous system. Module–trait relationships of filtered modules are shown in Supplementary Figure S4C. Correlation analysis presented three modules in brown, dark green, and lavender blush displayed the highest correlation between GS and MM (Figures 6A–C, cor > 0.4, *p* < 0.05). The top 1000 connections (according to connection weight) were used to construct a coexpression network of circRNAs in the brown and dark green modules (Figures 6D,E). The lavender blush 3 module included all 488 connections (Figure 6F). The highlighted network nodes probably constituted the core of the network (Figures 6D–F). In addition, the presynaptic active zone, microtubule cytoskeleton, and brain development were highly enriched (Figures 6G–I). When we calculated the correlation coefficient between 15 hub genes in these three modules, we found that they could be clustered into three parts, consistent with their module identities (Supplementary Figure S5). Results of GO analysis in pink, black, and green modules are also shown (Supplementary Figures S4D–I).

### Single-Cell Transcriptome Profiling of the Hippocampus Identified Different Nerve Cell Types and Gene Expression Signatures

**FIGURE 7:**
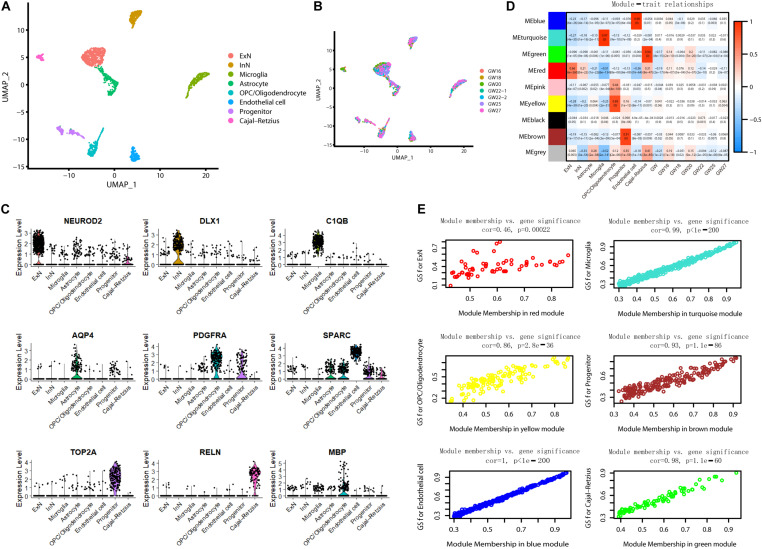
WGCNA for scRNA sequencing data. **(A)** Visualization of eight-cell types using UMAP. **(B)** Information of gestational week. **(C)** The violin plot shows the expression of representative genes in each cluster. **(D)** The correlation of module gene expression with cell type and gestational week. High relationships were only found between six modules with certain cell types (*r* > 0.6). **(E)** Module membership versus gene significance correlation in six modules showed high relationship with a single cell type (*r* > 0.6).

To further understand the molecular features in the hippocampus during brain development, we analyzed scRNA sequencing data for 2,054 fetal hippocampus cells obtained from GEO (GSE131258). We performed a uniform manifold approximation and projection (UMAP) analysis and classified cells into excitatory neurons (ExN), inhibitory neurons (InN), microglia, astrocytes, oligodendrocyte progenitor cells (OPCs)/oligodendrocytes, endothelial cells, progenitors, and Cajal Retzius cells (Zhong et al., 2020) (Figure 7A). Each cluster included cells from different gestational week tissues (Figure 7B). We calculated DE genes from each cluster and plotted representative DE genes (Zhong et al., 2020) (Figure 7C). We performed WGCNA to understand the gene coexpression relationships between different cell types. Notably, six out of nine coexpression modules showed a high correlation with single-cell type–specific expression (Figures 7D,E). Conversely, no module had an apparent relationship with any one of GW (*r* < 0.3).

### The Construction of the Core ceRNA Network

**FIGURE 8:**
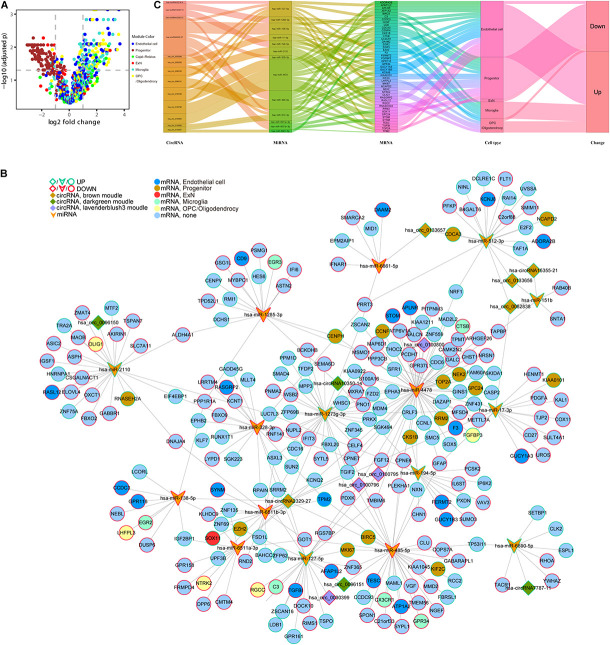
An integrative analysis of ceRNA regulation and cell-type information. **(A)** Different expression of cell type–specific module’s gene in DS. **(B)** Core network of 14 hub circRNAs, 16 DG miRNA targets, and 245 DE mRNA targets module information. **(C)** The Sankey diagram shows the flow of genes in 6 cell type–specific expression modules.

In the following step, we demonstrated the relationship between genes in six cell type–specific expression modules and their expression patterns in DS. The volcano plot shows that all DE genes in the brown module were downregulated and in the green module were upregulated. In addition, almost all of the DE genes in the blue, turquoise, and yellow modules were upregulated. Six and two memberships were upregulated and downregulated in the red module, respectively (Figure 8A and Supplementary Table S3). We then used the five hub circRNAs from brown, green, and lavender blush 3 modules, as well as 17 and 245 DE miRNA and mRNA targets to build a ceRNA network. Molecules that did not target any one of the DE mRNAs were deleted. The core ceRNA network is constructed of 14 circRNAs, 16 miRNAs, and 245 DE mRNAs (Figure 8B). The Sankey diagram highlights the flow-included genes in six cell type–specific expression modules (Figure 8C).

### The Convergent Functional Genomic (CFG) Score of Target Genes

AlzData provided multiple information of target genes, including the expression of the target gene regulated by AD genetic variants; significant physical interaction with APP, PSEN1, PSEN2, APOE, or MAPT; differentially expressed in AD mouse models before AD pathology emergence and the correlation of target gene expression with AD pathology in amyloid beta or tau line AD mouse models. The CFG score for the target gene was, thus, calculated (Table 3).

**TABLE 3:** CFG score of target genes.

Gene	eQTL	GWAS	PPI	Early_DEG	Pathology cor (abeta)	Pathology cor (tau)	CFG
*C3*	2	0	APP, PSEN1, PSEN2, MAPT	Yes	0.850, ***	0.761, ***	4
*NCAPD2*	41	0	MAPT	Yes	−0.090, ns	-0.801, ***	4
*ADORA2B*	7	0	–	Yes	0.725, ***	0.652, **	3
*ATP1A2*	1	0	–	Yes	0.460, **	-0.110, ns	3
*CD9*	2	0	–	Yes	0.873, ***	0.566, *	3
*CTSB*	NA	0	APP, APOE	Yes	0.801, ***	0.599, *	3
*DAAM2*	3	0	–	Yes	0.477, **	0.307, ns	3
*EGR2*	2	0	–	Yes	−0.376, *	−0.196, ns	3
*GPR116*	0	2	APP, PSEN1, PSEN2, MAPT, APOE	Yes	−0.045, ns	−0.023, ns	3
*GPR34*	1	NA	NA	Yes	0.852, ***	0.876, ***	3
*GUCY1A3*	3	0	–	yes	−0.191, ns	−0.729, **	3
*GUCY1B3*	1	0	–	yes	−0.246, ns	−0.581, *	3
*NTRK2*	1	0	APP, PSEN1, PSEN2	NA	0.543, ***	0.538, *	3
*CCDC3*	0	3	–	no	0.557, ***	0.824, ***	2
*CCNF*	1	0	PSEN1	NA	0.078, ns	−0.247, ns	2
*CX3CR1*	4	0	–	NA	0.832, ***	0.485, ns	2
*EGR3*	1	0	–	yes	−0.045, ns	0.474, ns	2
*EZH2*	4	0	PSEN2	NA	NA	NA	2
*FERMT2*	2	100	–	NA	NA	NA	2
*FGFBP3*	1	0	NA	NA	−0.642, ***	−0.251, ns	2
*KIAA0101*	1	5	–	NA	NA	NA	2
*NEK2*	2	0	MAPT	NA	NA	NA	2
*OLIG1*	3	0	–	yes	0.175, ns	−0.156, ns	2
*TGFBI*	0	0	APP, APOE	no	0.532, ***	0.426, ns	2
*TPM2*	1	0	APP	NA	0.049, ns	−0.420, ns	2
*APLNR*	NA	0	APP, PSEN1, APOE	NA	NA	NA	1
*BIRC5*	0	0	PSEN2, APOE	NA	−0.089, ns	0.058, ns	1
*CDCA3*	2	0	NA	NA	0.031, ns	0.025, ns	1
*CENPH*	0	0	PSEN2	NA	NA	NA	1
*CKS1B*	2	0	–	NA	NA	NA	1
*F3*	3	0	–	NA	NA	NA	1
*KCNJ8*	0	0	–	yes	0.028, ns	0.177, ns	1
*KIF2C*	0	0	PSEN1, PSEN2	NA	NA	NA	1
*RASGRP2*	2	0	–	NA	−0.262, ns	−0.200, ns	1
*RNASEH2A*	1	0	–	NA	NA	NA	1
*RRM2*	4	0	–	NA	−0.080, ns	−0.398, ns	1
*SOX11*	1	0	–	NA	NA	NA	1
*SPC24*	NA	0	PSEN2	NA	−0.030, ns	0.113, ns	1
*STOM*	1	0	–	NA	NA	NA	1
*TESC*	2	0	–	NA	−0.165, ns	0.376, ns	1
*TOP2A*	NA	0	–	Yes	0.065, ns	−0.229, ns	1
*AFAP1L2*	0	0	–	NA	NA	NA	0
*LHFPL3*	0	0	NA	NA	NA	NA	0
*MKI67*	0	0	–	NA	NA	NA	0
*RASL12*	0	0	–	NA	0.177, ns	0.291, ns	0
*RGCC*	NA	0	–	NA	NA	NA	0
*SYNM*	0	0	–	NA	NA	NA	0

eQTL, expression of target gene is regulated by AD genetic variants (genetic variants: IGAP GWAS *P*-val < 1E-3; eQTL: *P*-val < 1E-3). GWAS, IGAP P-val < 1E-3. PPI, target gene has significant physical interaction with APP, PSEN1, PSEN2, APOE or MAPT (*P*-val < 0.05). Early_DEG, target gene is differentially expressed in AD mouse models before AD pathology emergence. Pathology cor (abeta), correlation of target gene expression with AD pathology in abeta line AD mouse models (r, *P*-val; ns: *P*-val > 0.05; *: *P*-val < 0.05; **: *P*-val < 0.01; ***: *P*-val < 0.001). Pathology cor (tau), correlation of target gene expression with AD pathology in tau line AD mouse models (r, *P*-val; ns: *P*-val > 0.05; *: *P*-val < 0.05; **: *P*-val < 0.01; ***: *P*-val < 0.001). CFG, total CFG score of target gene, 1 CFG point is added if any of above evidence is significant, CFG point ranges from 0 to 5.

## Discussion

The circRNA–miRNA–mRNA regulatory network dysfunction represents an essential layer of epigenetic control in central nervous system disorders. Here, we report, for the first time, a systematic investigation of the expression profiles of circRNAs in the fetal hippocampus of DS. The expression profiling of miRNA and mRNA for DS from our previous study and publicly available scRNA sequencing data was integrated to analyze the cell type–specific expression patterns. A core ceRNA network integrating circRNA, miRNA, and mRNA was constructed.

Several pathways corresponding with linear mRNAs of dysregulated circRNAs were critically involved with the nervous system. For instance, alterations of Glutamate *N*-methyl-D-aspartate receptor (NMDAR) and mGluR signaling together with a general deficiency in dendritic spine structure were linked with DS (Lockrow et al., 2011; Huo et al., 2018). DS was associated with mild-to-severe intellectual disability originating from embryogenesis in the fetus (Roizen and Patterson, 2003). A gene–disease network-based analysis obtained “intellectual disability” and “Alzheimer’s disease” terms, consistent with the typical phenotypes lower intelligence and early-onset AD in DS patients (Ruparelia et al., 2013).

Several miRNAs identified to be associated with DS previously potentially interacted with DE circRNAs, which were characterized in the present study. Of note, all these miRNAs were upregulated, whereas their corresponding DE circRNAs may downregulate their corresponding mRNAs by competitively binding with such miRNAs. We showed that *EZH2* was a direct target of hsa-miR-138-5p and may involve in the neurological deficiency of DS patients (Shi et al., 2016). The present research further complemented the interactions between *hsa_circ_0078328* and *miR-138-5p*. *Hsa_circ_0078328*, as an RNA sponge, could competitively bind *miR-138-5p* with *EZH2*, which may be partly involved in the regulation network of *EZH2* and further affect hippocampus development. Recent studies indicated that altered inhibitory transmission caused by *GRIK1* [encoding GluR5 kainate receptor (KAR)] triplication was an important novel factor in cognitive deficits (e.g., memory impairment) in DS (Valbuena et al., 2019). Our circRNA-based analysis for *GRIK1* showed that multiple signaling pathways were implicated in neuropathogenesis. The axon guidance pathway was involved in the formation of neural circuitry through nervous system development, and abnormal axon guidance may contribute to the reduction of long-distance connectivity formation and other DS brain phenotypes (Jia et al., 2017). Pharmacological stimulation of BDNF signaling could rescue synaptic plasticity and memory deficits in Ts65Dn mice (Parrini et al., 2017). The AD-related gene *APBB1* participates in synaptic plasticity, acquisition/retention for certain forms of memory formation, hippocampus-dependent learning, and long-term potentiation (Wang et al., 2009). *APBB1IP*, as an interacting protein of APBB1, was also reported to play a certain role in late-onset AD (Jungke et al., 2011).

We constructed coexpression networks of DE circRNAs using WGCNA. GO analysis on the associated coding genes of circRNAs in each module was performed to unravel the underlying mechanisms. Both the brown and lavender blush 3 modules were enriched in synapse-related pathways. Moreover, the lavender blush 3 module was also enriched in embryo/head/brain development. The dark green module is associated with the maintenance of RNA splicing and microtubule-organizing, implicated in neurodegeneration (Ivashko-Pachima et al., 2017; Hsieh et al., 2019). WGCNA for scRNA identified six modules with obvious cell type–specific expression patterns. In addition, these six modules also tend to be stable through the covered developmental stage, implying that such a stable expression might be essential for hippocampus development. Different expression patterns of these six modules’ genes in DS may deserve attention. Interestingly, all DE genes in the brown module associated with the progenitor were downregulated. Previous reports suggested that the fetal neural stem/progenitor proliferation in nervous tissues was markedly impaired, leading to the neurocognitive DS features (Liu et al., 2015). However, most DE genes in the green, blue, turquoise, and yellow modules were upregulated, underlining the distinct dysfunction features and pathogenesis mechanism in different cell types. Therefore, neurology research under cellular resolution is urgent to capture the unbiased and systematic characterization of nerve cells in DS patients.

Molecules with known functions implicated in the nervous system were identified in the constructed core network, e.g., miR-138-*EZH2* (Shi et al., 2016). Previous studies showed that Olig1 triplication led to interneuron overproduction, which was responsible for the imbalance between excitatory and inhibitory neurons and increased inhibitory. The widespread effect of Olig1 and Olig2, e.g., overexpression from the early developmental stage, was the main component of the cognitive deficit phenotype in DS (Chakrabarti et al., 2010). Cathepsin B (encoded by *CTSB*) in secretory vesicles was thought to function as a β-secretase, producing neurotoxic β-amyloid (Aβ) peptides. The regulated secretory vesicles produced the majority of extracellular β-amyloid of AD, participating as a notable factor in the severe memory loss in AD (Hook et al., 2012). *TPM2* is essential for memory formation. The knockdown of *TPM2* impairs spatial learning and memory in rats (Hsu et al., 2017). In addition, variants in *FERMT* (rs17125944), *NCAPD2* (rs740850), and *TESC* (rs7294919) were associated with brain amyloidosis (Apostolova et al., 2018), AD (Lee et al., 2008), and hippocampal volume/brain development (Stein et al., 2012). Our research also revealed the epigenetic alteration of these genes. In addition, multiple genes, e.g., *C3, NCAPD2*, and *ADORA2B*, were related to molecular mechanisms underlying AD. The specific function of molecules in the core network needs further investigation.

There are several limitations to the present study. First, the scarcity of samples makes it difficult to enhance the representativeness of the qRT-PCR experiment. More accurate identification results of DE circRNAs need to be validated in a larger cohort. Second, DE circRNAs, miRNAs, and mRNAs are not identified in the same cohort, which may disturb the functional regulatory network construction and pathway enrichment underlying DS. Third, the expression of circRNAs across other areas of the brain need comprehensive analysis. Finally, the molecular mechanisms of hsa_circ_0078328/miR-138-5p/*EZH2* and circ-GRIK1s need more investigation.

## Conclusion

Our study represents an integrated analysis of the expression profiles of the circRNAs and ceRNA network in the hippocampus of DS patients. Our findings may provide implications in further research for the novel DE circRNAs as miRNA sponges and their potential molecular biological functions in the development and treatment for patients with chronic neurodegenerative diseases or intellectual disability.

## Data Availability Statement

Single-Cell RNA (scRNA) sequencing data from human fetal hippocampus is available from the Gene Expression Omnibus (GEO) (http://www.ncbi.nlm.nih.gov/geo/) under the accession number GSE131258.

## Ethics Statement

The studies involving human participants were reviewed and approved by the Human Ethics Committee of the People’s Hospital of Zhengzhou University. The patients/participants provided their written informed consent to participate in this study.

## Author Contributions

SL, KD, and BH designed the research. LQ, WS, SB, ZW, QW, XW, and JG performed the data collection. SW, XT, KD, SL, and BH analyzed the data. SW wrote the manuscript. KD, XT, SL, BH, and JG revised the manuscript critically. All authors read and approved the final manuscript.

## Conflict of Interest

The authors declare that the research was conducted in the absence of any commercial or financial relationships that could be construed as a potential conflict of interest.

## Footnotes

1 http://www.ncbi.nlm.nih.gov/geo/

2 http://starbase.sysu.edu.cn/

3 www.alzdata.org

## Supplementary Material

Supplementary Figure 1 A pipeline for bioinformatic analysis.

Supplementary Figure 2 Quality control for the microarray data. **(A)** A scatter plot shows the expressional variation of circRNAs in the fetal hippocampal tissues of DS patients versus the controls. The upper red points indicate circRNAs with a fold change greater than 2.0, and the lower green points indicate that less than 2.0. **(B)** A volcano plot shows the DE circRNAs. Dots in different colors show different fold changes. Red, fold change > 2; blue: fold change < −2. The horizontal line indicates the *p*-value of 0.05. **(C)** A box plot shows a similar distribution of circRNAs for the samples after normalization.

Supplementary Figure 3 A network of circRNA–miRNA interaction of DE circRNAs was used for qRT-PCR verification. Pink rounds represent circRNAs, and the blue are their potential miRNA targets with no difference of expression, yellow rounds represent upregulated potential miRNA targets.

Supplementary Figure 4 WGCNA for circRNA. The correlation of module membership and gene significance in the pink **(A)**, black **(B)**, and green **(C)** modules. The coexpression network of the pink **(D)**, black **(E)**, and green **(F)** module with 5 hub nodes’ circRNA shown in each module. GO enrichment analysis for the pink **(G)**, black **(H)**, and green **(I)** module.

Supplementary Figure 5 The correlation between 15 hub genes from the brown, dark green, and lavender blush 3 modules.

Supplementary Table 1 Primers for validation.

Supplementary Table 2 DE circRNAs with adjusted *p* (0.01 and log2|FC|> 1.

Supplementary Table 3 The expression of genes shown in Figure 8A.
