# Supplementary figures and images for: Integrative Analysis Extracts a Core ceRNA Network of the Fetal Hippocampus With Down Syndrome
Source: Front Genet. 2020 Nov 30;11:565955. doi: 10.3389/fgene.2020.565955 (PMC7735064; doi:10.3389/fgene.2020.565955)

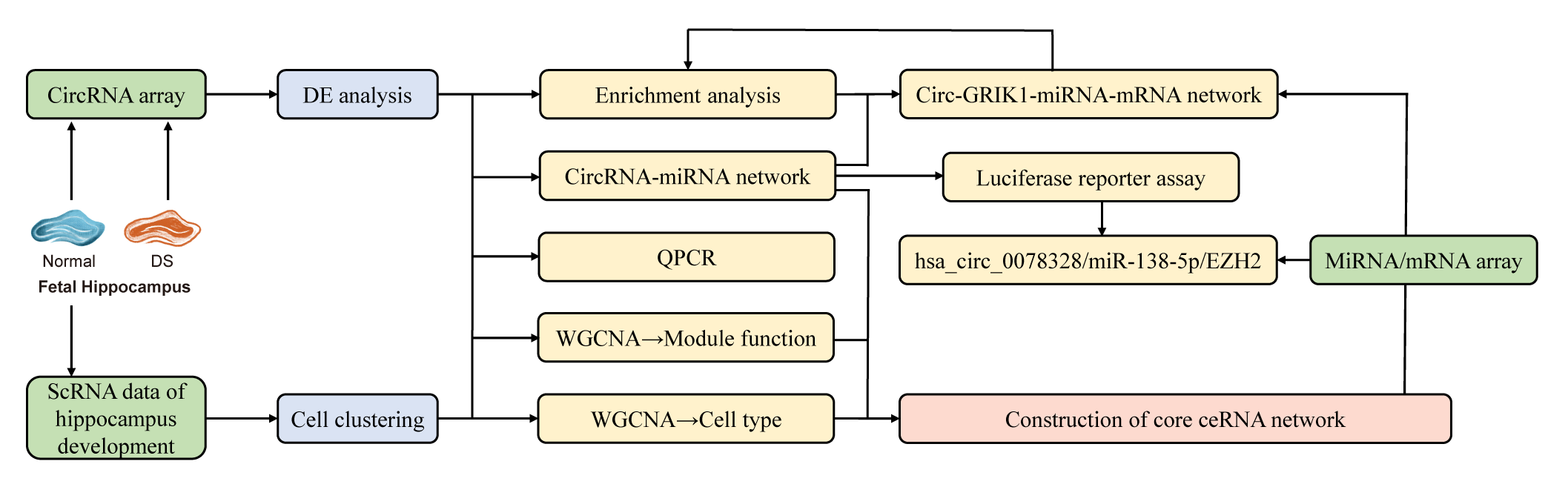

Supplement: Supplementary Figure 1 — A pipeline for bioinformatic analysis. [file Image_1.TIF]

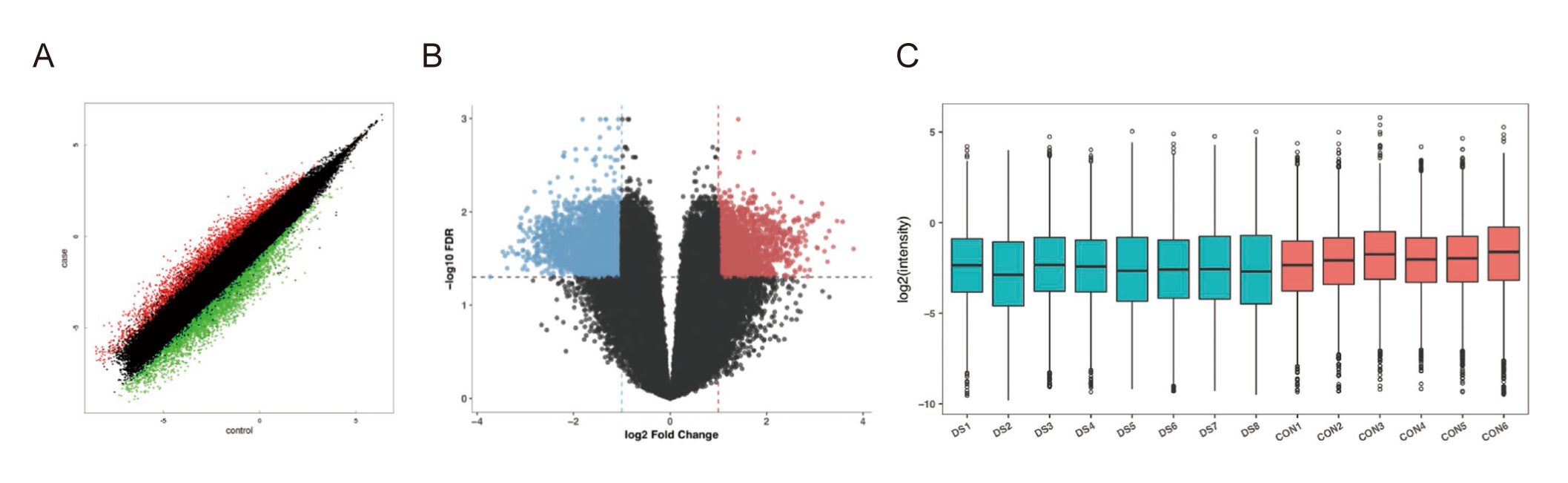

Supplement: Supplementary Figure 2 — Quality control for the microarray data. (A) A scatter plot shows the expressional variation of circRNAs in the fetal hippocampal tissues of DS patients versus the controls. The upper red points indicate circRNAs with a fold change greater than 2.0, and the lower green points indicate that less than 2.0. (B) A volcano plot shows the DE circRNAs. Dots in different colors show different fold changes. Red, fold change > 2; blue: fold change < −2. The horizontal line indicates the p-value of 0.05. (C) A box plot shows a similar distribution of circRNAs for the samples after normalization. [file Image_2.TIF]

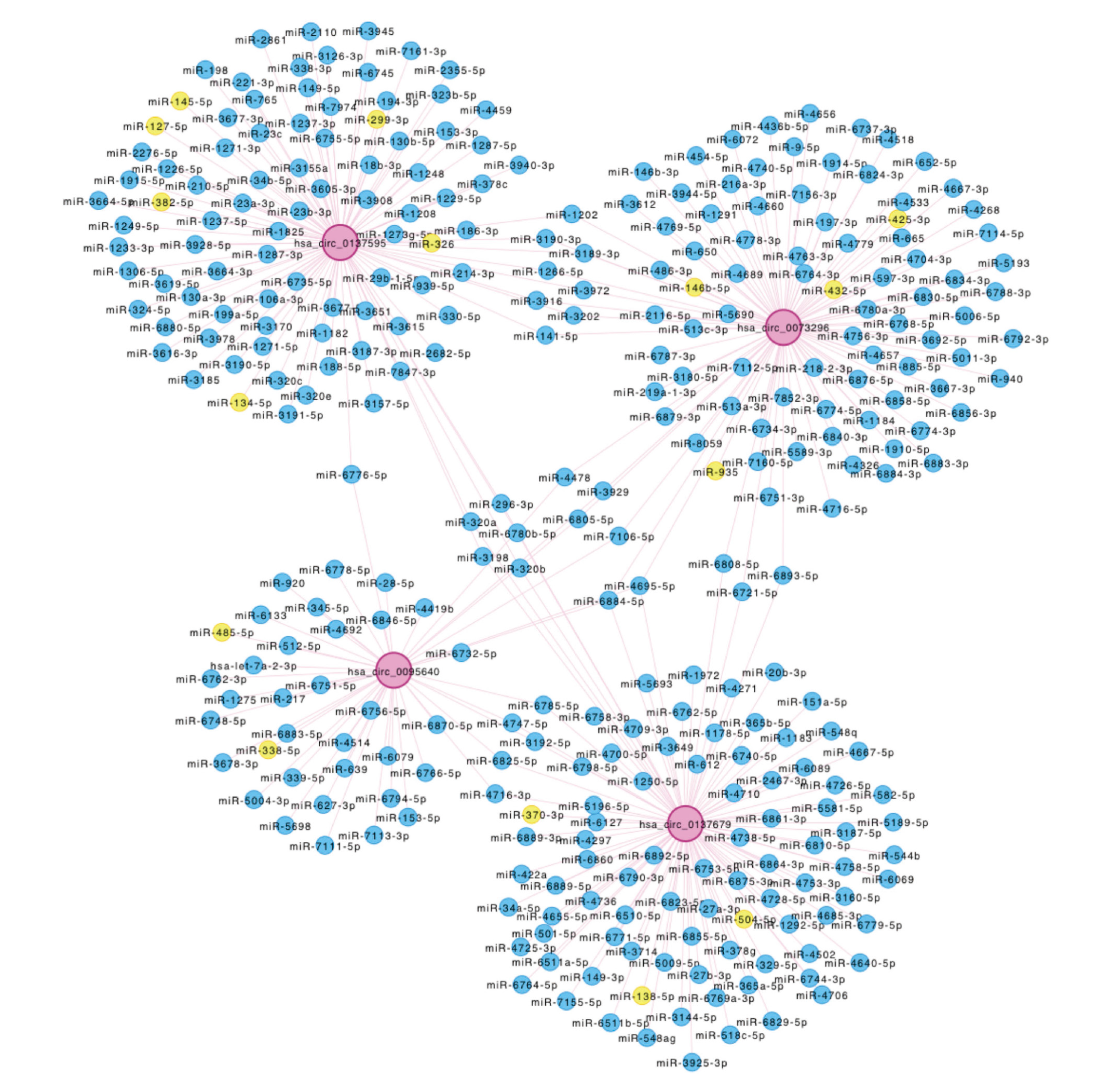

Supplement: Supplementary Figure 3 — A network of circRNA–miRNA interaction of DE circRNAs was used for qRT-PCR verification. Pink rounds represent circRNAs, and the blue are their potential miRNA targets with no difference of expression, yellow rounds represent upregulated potential miRNA targets. [file Image_3.TIF]

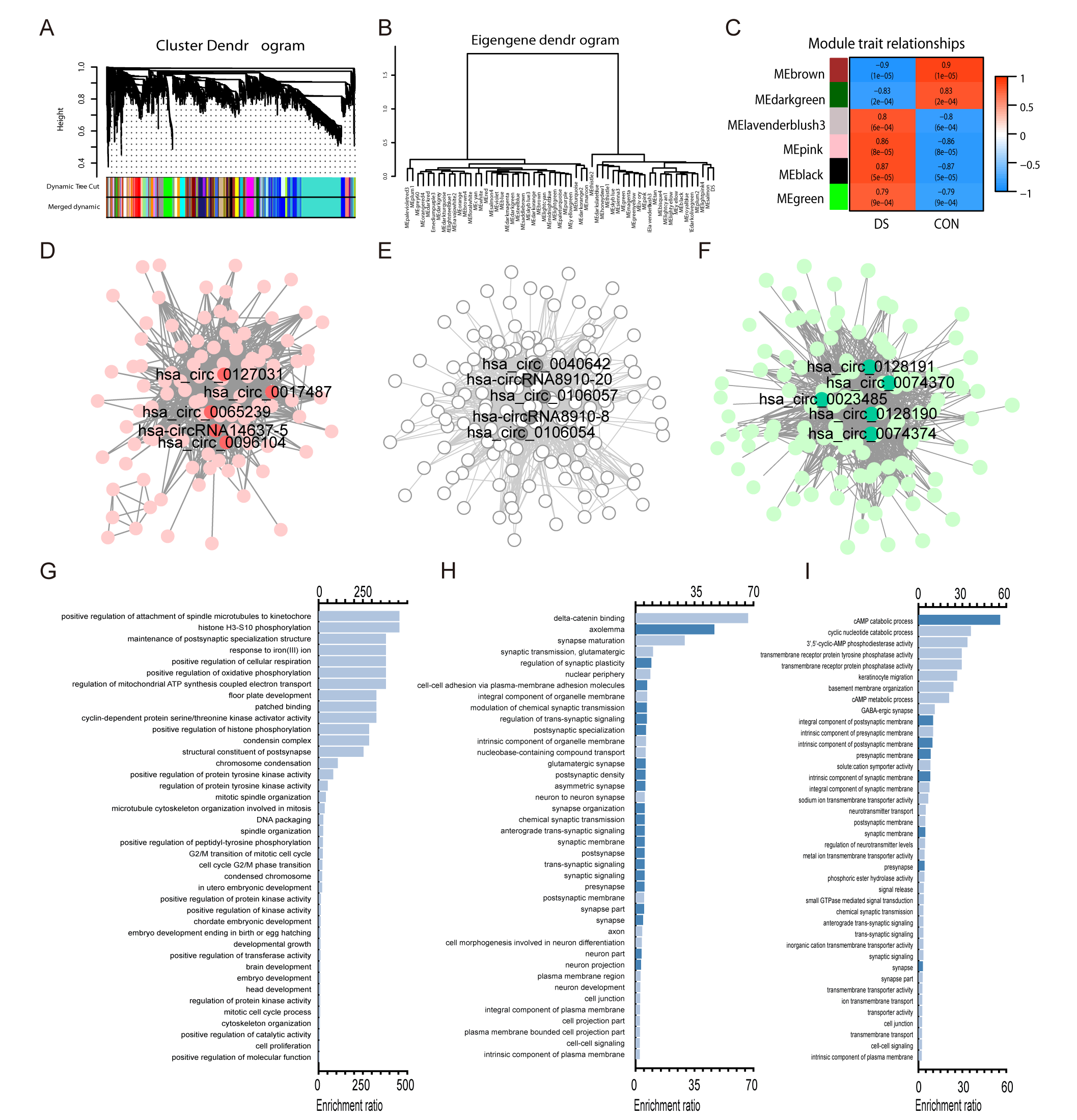

Supplement: Supplementary Figure 4 — WGCNA for circRNA. The correlation of module membership and gene significance in the pink (A), black (B), and green (C) modules. The coexpression network of the pink (D), black (E), and green (F) module with 5 hub nodes’ circRNA shown in each module. GO enrichment analysis for the pink (G), black (H), and green (I) module. [file Image_4.tif]

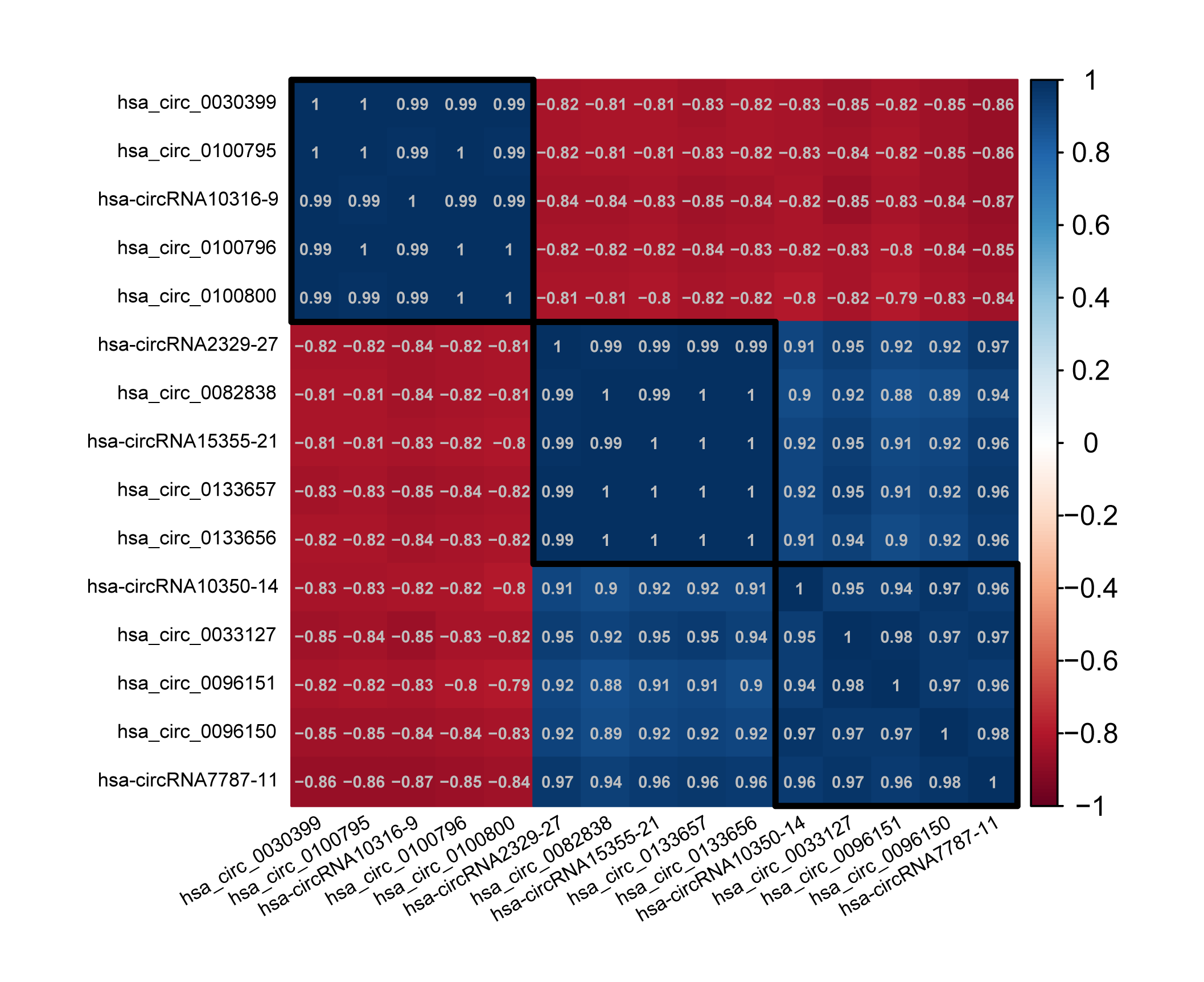

Supplement: Supplementary Figure 5 — The correlation between 15 hub genes from the brown, dark green, and lavender blush 3 modules. [file Image_5.TIF]
