# Supplementary material for: Integrative Analysis Extracts a Core ceRNA Network of the Fetal Hippocampus With Down Syndrome
Source: Front Genet. 2020 Nov 30;11:565955. doi: 10.3389/fgene.2020.565955 (PMC7735064; doi:10.3389/fgene.2020.565955)
Supplement: Supplementary Table 1 — Primers for validation. [file Table_1.PDF]

Supplementary Table 1. Primers for validation

Gene	Primer sequences
β-actin	F:5'CCTGTACGCCAACACAGTGC3'
	R:5'ATACTCCTGCTTGCTGATCC3'
hsa_circ_0137595	F:5' GCTTTCTGGAATACATGACCCC 3'
	R:5' GGATTGAGACAGTGACATCAGC 3'
hsa_circ_0095640	F:5' ACTGCCTGTGACTCTCAAGA 3'
	R:5' CTCCCTGTCAAGAGCTGCT 3'
hsa_circ_0137679	F:5' AGGGTGGAAAGGAGGAATCA 3'
	R:5' CTAAGCACTGGGGAAGGGAG 3'
hsa_circ_0073296	F:5' AAGTGGTAGTGGTGATGGGG 3'
	R:5' GGTTGATCCCTCCTCTGCAA 3'
hsa_circ_0023500	F:5'AGTTCTGCTGCCGAGACTAA 3'
	R:5'GCACCACCTCGTAGTAGTCT 3'
hsa_circ_0115770	F:5'GACCGCCCTGGTAAGAAACA 3'
	R:5'TGAAACCCAGGATGTTTGACA 3'
hsa_circ_0115774	F:5'CAGCTGCCCTCTGGGAATAA 3'
	R:5'TGTGGTGTTAGGCATCAGGG 3'
hsa_circ_0115775	F:5'ACCTTTACCCAGATTATGCAGCT 3'
	R:5'TGTTTCAAAAATCCCTCCTGTG 3'
