# Supplementary material for: Integrative Analysis Extracts a Core ceRNA Network of the Fetal Hippocampus With Down Syndrome
Source: Front Genet. 2020 Nov 30;11:565955. doi: 10.3389/fgene.2020.565955 (PMC7735064; doi:10.3389/fgene.2020.565955)
Supplement: Supplementary Table 2 — DE circRNAs with adjusted p (0.01 and log2|FC|> 1. [file Table_2.PDF]

Supplementary Table 2. DE circRNAs with adjusted *p* < 0.01 and log2FC > 1

CircRNA ID	Adjusted p	Log2FC
hsa_circ_0061485	0.001021662	1.412726524
hsa-circRNA14604-27	0.002130076	1.178767214
hsa_circ_0041459	0.00230515	1.738066308
hsa_circ_0078328	0.00230515	1.441704251
hsa_circ_0003068	0.002607779	1.420635452
hsa_circ_0075691	0.003830116	1.027389166
hsa_circ_0131090	0.004742799	2.070113844
hsa_circ_0022409	0.005310215	1.399036644
hsa_circ_0091475	0.005868303	1.707250928
hsa_circ_0092572	0.005868303	1.002954398
hsa-circRNA9513-15	0.005918597	1.230817146
hsa-circRNA7492-17	0.006214601	1.124468803
hsa-circRNA8910-13	0.006336777	1.528395062
hsa_circ_0139724	0.006336777	1.143184038
hsa-circRNA11308-1	0.006455856	2.146535176
hsa_circ_0097880	0.006866531	1.723560487
hsa_circ_0056326	0.006892448	1.246762234
hsa_circ_0040963	0.006893543	2.539111776
hsa_circ_0140138	0.006893543	1.773699138
hsa_circ_0049261	0.006893543	1.733068061
hsa_circ_0098211	0.006893543	1.27289073
hsa_circ_0115879	0.006893543	1.221525774
hsa_circ_0016132	0.006893543	1.062589668
hsa_circ_0022404	0.007042603	1.66953232
hsa_circ_0070160	0.007042603	1.012001881
hsa_circ_0040960	0.007200993	1.761229273
hsa-circRNA13240-4	0.007200993	1.165651323
hsa-circRNA13173-2	0.007310004	1.512489314
hsa_circ_0055073	0.007546918	1.035571859
hsa-circRNA12268-5	0.007593606	1.628720634
hsa_circ_0040300	0.007696966	1.154627127
hsa_circ_0107483	0.007874846	2.71817904
hsa_circ_0137079	0.007874846	2.462710088
hsa-circRNA13198-9	0.007874846	2.27031162
hsa_circ_0139713	0.007874846	1.717192853
hsa_circ_0070159	0.007874846	1.261335285
hsa_circ_0074859	0.007874846	1.180302777
hsa_circ_0043870	0.007874846	1.173253342
hsa_circ_0040304	0.007874846	1.141615357
hsa_circ_0139988	0.007874846	1.121558575
hsa_circ_0092570	0.007874846	1.096257994
hsa_circ_0070868	0.007874846	1.032190403

hsa_circ_0109005	0.007995676	1.730643671
hsa-circRNA6034-41	0.007995676	1.099726798
hsa_circ_0131097	0.00800285	1.677977894
hsa-circRNA2911-12	0.008032385	1.693444335
hsa_circ_0139158	0.008032385	1.607433115
hsa_circ_0016135	0.008038467	1.174499075
hsa_circ_0131237	0.00813466	3.149688551
hsa_circ_0074688	0.008169159	1.619448268
hsa_circ_0118909	0.008169159	1.10711613
hsa_circ_0040966	0.008195715	2.05303921
hsa_circ_0115842	0.008275819	1.926115401
hsa_circ_0110582	0.008283504	1.049715641
hsa_circ_0040308	0.008283504	1.038191355
hsa_circ_0040640	0.008346361	1.075441641
hsa_circ_0115886	0.008645576	1.317154528
hsa_circ_0061812	0.008647915	2.003223578
hsa_circ_0135479	0.008647915	1.898654169
hsa_circ_0016687	0.008647915	1.185826073
hsa_circ_0028005	0.008647915	1.045405309
hsa-circRNA11475	0.008782486	1.418983831
hsa_circ_0139991	0.008782486	1.130423702
hsa_circ_0055868	0.008782486	1.067696934
hsa_circ_0139157	0.008873312	1.06331021
hsa_circ_0061573	0.008879181	1.429780748
hsa_circ_0003582	0.008879181	1.422426569
hsa_circ_0139989	0.008893717	1.481041512
hsa_circ_0034352	0.008965225	1.066733737
hsa_circ_0115880	0.009119007	1.073240326
hsa_circ_0128829	0.009127039	2.282296644
hsa_circ_0111744	0.009162855	1.503289314
hsa_circ_0140135	0.009183578	1.797334369
hsa_circ_0085807	0.009202898	1.084915682
hsa_circ_0097887	0.009206437	1.896854313
hsa_circ_0016130	0.009206437	1.605354002
hsa_circ_0114469	0.009206437	1.301023823
hsa_circ_0038646	0.009206437	1.289275624
hsa_circ_0089967	0.009206437	1.208498277
hsa_circ_0032933	0.009206437	1.00953848
hsa-circRNA6034-101	0.009237381	1.288544112
hsa_circ_0062997	0.009237381	1.216925113
hsa_circ_0128193	0.009256283	1.079601271
hsa_circ_0101906	0.009266431	1.779419903
hsa-circRNA9513-12	0.009266431	1.556570732
hsa-circRNA10482-6	0.009332713	3.061875092

hsa-circRNA8910-8	0.009332713	2.04530114
hsa_circ_0138933	0.009332713	1.571496165
hsa-circRNA4561-1	0.009332713	1.21503743
hsa-circRNA7949-13	0.009332713	1.053150012
hsa_circ_0041430	0.009332713	1.051483199
hsa_circ_0074624	0.009332713	1.038603148
hsa_circ_0111658	0.009332713	1.005242962
hsa_circ_0130945	0.009395988	1.57400241
hsa_circ_0110045	0.009395988	1.161417504
hsa-circRNA6034-75	0.009395988	1.127075737
hsa_circ_0049260	0.009535046	1.519056133
hsa_circ_0139715	0.009535046	1.426103103
hsa_circ_0028523	0.009535046	1.210904094
hsa_circ_0061484	0.009570483	1.412877162
hsa_circ_0098505	0.009570483	1.146793864
hsa-circRNA15014-1	0.009586053	2.141376712
hsa_circ_0131100	0.009716396	1.607709196
hsa_circ_0038640	0.009716396	1.395463162
hsa_circ_0041436	0.009716396	1.13810775
hsa_circ_0099340	0.009716396	1.136229973
hsa_circ_0112000	0.009761388	1.56492796
hsa_circ_0065938	0.009761388	1.084866601
hsa_circ_0017646	0.009762649	1.441026432
hsa_circ_0115002	0.009956307	1.459126027
hsa_circ_0093371	0.009971684	2.180568837
hsa_circ_0136733	0.001021662	-1.814926364
hsa-circRNA8010-1	0.001021662	-1.446704261
hsa_circ_0090502	0.001021662	-1.33181098
hsa_circ_0005095	0.001021662	-1.327668677
hsa-circRNA7988-39	0.001021662	-1.071559143
hsa_circ_0090327	0.00126852	-1.259032632
hsa_circ_0090324	0.00126852	-1.070522386
hsa-circRNA8010-3	0.001594677	-1.451591741
hsa_circ_0095418	0.002030762	-1.06352876
hsa_circ_0056745	0.002128319	-1.15738147
hsa_circ_0104650	0.002130076	-1.738652176
hsa_circ_0090503	0.002130076	-1.462741688
hsa_circ_0136735	0.00214305	-2.181936884
hsa_circ_0136728	0.00214305	-1.955535261
hsa_circ_0098616	0.00214305	-1.121983394
hsa_circ_0136730	0.002319914	-2.186236454
hsa_circ_0136725	0.002529992	-2.215619925
hsa_circ_0136729	0.002566874	-2.196673459
hsa_circ_0090506	0.002566874	-1.340968594

hsa_circ_0027606	0.002607779	-1.632040163
hsa_circ_0008274	0.002607779	-1.541978098
hsa_circ_0021339	0.002607779	-1.236631696
hsa_circ_0113752	0.002794473	-1.941440714
hsa_circ_0000277	0.002794473	-1.591628167
hsa_circ_0136732	0.002794473	-1.56494558
hsa_circ_0126619	0.002794473	-1.148561138
hsa-circRNA2424-13	0.002794473	-1.084841805
hsa_circ_0122211	0.003155175	-1.543926651
hsa_circ_0099088	0.003343111	-1.222831013
hsa_circ_0090325	0.003830116	-1.153737539
hsa_circ_0099091	0.004021667	-1.317409757
hsa_circ_0104649	0.004274365	-2.032663284
hsa_circ_0138306	0.004280906	-2.325721969
hsa_circ_0001278	0.004649243	-1.156465697
hsa_circ_0035653	0.004697364	-1.573850712
hsa_circ_0090505	0.004697364	-1.498163688
hsa_circ_0101528	0.00490069	-2.490637506
hsa_circ_0136727	0.005310215	-1.514715179
hsa_circ_0113755	0.005639256	-2.017579124
hsa_circ_0008399	0.005639256	-1.570840335
hsa_circ_0090326	0.005639256	-1.024299873
hsa_circ_0113751	0.005666244	-1.706655215
hsa_circ_0059599	0.005666244	-1.064018227
hsa_circ_0103037	0.005826033	-1.919929457
hsa_circ_0121010	0.005826033	-1.462105677
hsa_circ_0039706	0.005868303	-1.55047861
hsa_circ_0099086	0.005868303	-1.170334983
hsa_circ_0101529	0.006112014	-2.810070164
hsa-circRNA2246-5	0.006214601	-2.474816934
hsa_circ_0107143	0.006336777	-1.174798144
hsa_circ_0091053	0.006435193	-1.144190454
hsa_circ_0101530	0.006866531	-2.298665726
hsa_circ_0136731	0.006866531	-1.612180228
hsa_circ_0030008	0.006866531	-1.366383803
hsa-circRNA3199-2	0.006866531	-1.076280235
hsa_circ_0006010	0.006871291	-1.972068582
hsa-circRNA15085-7	0.006871291	-1.660434448
hsa_circ_0095420	0.006871291	-1.126421258
hsa_circ_0125921	0.006893543	-1.280933623
hsa-circRNA16025-4	0.006962932	-1.354877491
hsa-circRNA15699-14	0.007042603	-1.214580728
hsa_circ_0044697	0.007042603	-1.087613317
hsa_circ_0010877	0.007106913	-1.033173313

hsa_circ_0130501	0.007200993	-1.686920101
hsa_circ_0035205	0.007200993	-1.398196294
hsa-circRNA1872-31	0.007200993	-1.284911733
hsa_circ_0070036	0.007310004	-1.187724017
hsa_circ_0065249	0.007310004	-1.091712058
hsa_circ_0130020	0.007431099	-2.493496014
hsa_circ_0079786	0.007431099	-1.735182745
hsa-circRNA949-20	0.007431099	-1.46919054
hsa-circRNA14035-1	0.007431099	-1.12998422
hsa_circ_0085615	0.007534158	-2.159167494
hsa_circ_0114833	0.007696966	-1.039454029
hsa_circ_0027824	0.007874846	-2.418401539
hsa_circ_0068091	0.007874846	-2.330094969
hsa_circ_0033134	0.007874846	-2.000968763
hsa_circ_0103036	0.007874846	-1.875072162
hsa_circ_0007020	0.007874846	-1.801070959
hsa_circ_0008002	0.007874846	-1.741440214
hsa_circ_0033143	0.007874846	-1.577027431
hsa_circ_0071680	0.007874846	-1.55606843
hsa_circ_0104117	0.007874846	-1.536184905
hsa_circ_0016649	0.007874846	-1.491087657
hsa_circ_0002138	0.007874846	-1.48079296
hsa_circ_0035649	0.007874846	-1.477856205
hsa_circ_0104110	0.007874846	-1.472138236
hsa_circ_0092388	0.007874846	-1.41270259
hsa_circ_0069536	0.007874846	-1.408074925
hsa_circ_0035648	0.007874846	-1.387705054
hsa_circ_0123480	0.007874846	-1.342637286
hsa_circ_0009010	0.007874846	-1.311596334
hsa_circ_0098470	0.007874846	-1.252938027
hsa-circRNA1623-6	0.007874846	-1.250246507
hsa_circ_0115287	0.007874846	-1.230481479
hsa_circ_0012245	0.007874846	-1.173629236
hsa-circRNA11180-24	0.007874846	-1.063562317
hsa_circ_0117683	0.007874846	-1.02051867
hsa_circ_0099084	0.007881963	-1.340534676
hsa_circ_0004866	0.007881963	-1.240277588
hsa-circRNA1244-16	0.007960702	-1.211664259
hsa_circ_0134130	0.007995676	-1.545023526
hsa_circ_0070042	0.007995676	-1.108825485
hsa_circ_0040820	0.007995676	-1.006218544
hsa_circ_0033137	0.00800285	-2.110628736
hsa_circ_0079785	0.008032385	-1.367142779
hsa-circRNA10154-2	0.008032385	-1.173370615

hsa_circ_0033139	0.008038467	-1.840252943
hsa_circ_0054673	0.008074535	-1.210751178
hsa-circRNA2424-19	0.00813466	-2.193933617
hsa_circ_0086368	0.00813466	-2.009000993
hsa_circ_0135626	0.00813466	-1.896329677
hsa_circ_0008233	0.00813466	-1.81788488
hsa_circ_0112241	0.00813466	-1.544801607
hsa_circ_0047581	0.00813466	-1.228942286
hsa_circ_0025956	0.00813466	-1.203399323
hsa_circ_0099095	0.00813466	-1.155403103
hsa_circ_0081399	0.008169159	-2.09413467
hsa-circRNA8960-32	0.008169159	-1.105411746
hsa_circ_0002359	0.008182953	-1.577685004
hsa_circ_0111107	0.008246238	-1.684061374
hsa-circRNA2609-18	0.008275819	-1.327884512
hsa_circ_0086447	0.008337814	-1.152447283
hsa_circ_0005997	0.008533053	-1.661332936
hsa_circ_0079828	0.008555546	-1.679290472
hsa_circ_0122212	0.008555546	-1.38239734
hsa_circ_0099081	0.008555546	-1.220188518
hsa_circ_0085437	0.008602595	-1.74857633
hsa_circ_0116382	0.008647915	-2.387868463
hsa_circ_0059375	0.008647915	-2.32723884
hsa-circRNA15355-9	0.008647915	-2.189779681
hsa-circRNA15691-20	0.008647915	-1.862922759
hsa_circ_0079837	0.008647915	-1.768710244
hsa_circ_0138343	0.008647915	-1.654410754
hsa_circ_0071685	0.008647915	-1.553110331
hsa_circ_0007548	0.008647915	-1.512507497
hsa_circ_0105442	0.008647915	-1.491000553
hsa_circ_0122216	0.008647915	-1.472519716
hsa-circRNA2609-15	0.008647915	-1.41595039
hsa_circ_0003501	0.008647915	-1.339477374
hsa-circRNA1872-1	0.008647915	-1.276848605
hsa_circ_0070031	0.008647915	-1.268128011
hsa_circ_0079598	0.008647915	-1.186192581
hsa_circ_0003914	0.008647915	-1.183628333
hsa_circ_0138111	0.008647915	-1.15100121
hsa_circ_0065304	0.008647915	-1.088744763
hsa_circ_0001651	0.008647915	-1.074752451
hsa_circ_0027478	0.008647915	-1.057671715
hsa_circ_0008304	0.00869019	-1.738119588
hsa_circ_0027481	0.00869019	-1.096639086
hsa-circRNA9533-14	0.00869019	-1.086748859

hsa_circ_0005325	0.00869019	-1.017393482
hsa_circ_0138308	0.008782486	-2.761559923
hsa_circ_0006225	0.008782486	-2.100040643
hsa-circRNA15085-20	0.008782486	-1.649745933
hsa_circ_0113806	0.008782486	-1.571333679
hsa-circRNA6201-6	0.008782486	-1.550665937
hsa_circ_0138733	0.008782486	-1.161618262
hsa_circ_0025950	0.008782486	-1.158168045
hsa_circ_0021340	0.008782486	-1.003704525
hsa_circ_0031639	0.008785989	-1.928875266
hsa_circ_0044399	0.008873312	-2.395734033
hsa_circ_0079591	0.008873312	-1.197237673
hsa_circ_0031781	0.008879181	-1.88545332
hsa_circ_0134132	0.008879181	-1.766906359
hsa-circRNA2609-6	0.008893717	-1.319521286
hsa_circ_0002551	0.008893717	-1.24319088
hsa-circRNA8444-4	0.008893717	-1.129989387
hsa_circ_0137212	0.008893717	-1.12203867
hsa_circ_0112440	0.008941197	-1.543596723
hsa_circ_0113754	0.008941882	-2.03097674
hsa_circ_0035656	0.008965225	-1.325141417
hsa_circ_0116385	0.009011568	-2.491757664
hsa_circ_0044401	0.009038758	-2.954673304
hsa_circ_0090504	0.009058124	-1.204195404
hsa-circRNA1765-2	0.009058124	-1.038923019
hsa_circ_0038228	0.009119007	-1.012774257
hsa_circ_0001009	0.009127039	-1.563113672
hsa_circ_0054676	0.009127039	-1.386464034
hsa_circ_0103746	0.009127039	-1.258198839
hsa-circRNA10441-31	0.009162855	-2.003637825
hsa-circRNA9660-3	0.009162855	-1.972807329
hsa_circ_0004904	0.009162855	-1.97049628
hsa_circ_0103038	0.009162855	-1.914481083
hsa_circ_0019775	0.009162855	-1.243031907
hsa_circ_0016562	0.009162855	-1.116830308
hsa_circ_0101742	0.009162855	-1.0670759
hsa_circ_0079842	0.009183578	-1.695679683
hsa_circ_0008639	0.009183578	-1.268252642
hsa_circ_0122206	0.009206437	-1.859956759
hsa_circ_0008153	0.009206437	-1.772961476
hsa_circ_0126303	0.009206437	-1.38100062
hsa_circ_0103748	0.009206437	-1.285411426
hsa-circRNA13518-114	0.009206437	-1.254120653
hsa_circ_0120389	0.009206437	-1.052911177

hsa_circ_0126287	0.009206437	-1.014042776
hsa_circ_0134200	0.009237381	-1.692883414
hsa_circ_0033141	0.009237381	-1.554565034
hsa_circ_0020257	0.009256283	-1.428519849
hsa_circ_0086369	0.009266431	-1.70360274
hsa_circ_0016707	0.009266431	-1.703063876
hsa_circ_0134138	0.009266431	-1.6240792
hsa_circ_0081401	0.009271876	-2.113695505
hsa_circ_0044388	0.009332713	-3.025358671
hsa_circ_0098173	0.009332713	-1.926566267
hsa_circ_0086444	0.009332713	-1.10203019
hsa_circ_0036563	0.009332713	-1.018334644
hsa_circ_0071682	0.009333094	-1.910573354
hsa_circ_0068092	0.009372128	-2.169746867
hsa_circ_0104118	0.009395988	-1.416955897
hsa_circ_0102291	0.009395988	-1.329058287
hsa-circRNA9426-3	0.009395988	-1.29385154
hsa_circ_0018137	0.009395988	-1.246890239
hsa-circRNA4955-1	0.009395988	-1.150111162
hsa_circ_0081740	0.009395988	-1.144792258
hsa-circRNA12576-2	0.009395988	-1.019221053
hsa-circRNA2935-3	0.009395988	-1.017462308
hsa_circ_0138299	0.009405727	-2.160671561
hsa_circ_0007308	0.009406064	-1.382099474
hsa-circRNA2033-27	0.009535046	-1.862782546
hsa_circ_0023986	0.009535046	-1.612309929
hsa_circ_0123946	0.009535046	-1.201894601
hsa_circ_0027484	0.009535046	-1.140499319
hsa_circ_0000225	0.009570483	-1.563276773
hsa_circ_0004116	0.009570483	-1.051611351
hsa-circRNA7231-9	0.009570483	-1.001177046
hsa_circ_0068093	0.009586053	-2.043919584
hsa_circ_0069508	0.009655535	-1.293371608
hsa-circRNA7836-89	0.009655535	-1.188003001
hsa_circ_0131018	0.009655535	-1.150751142
hsa_circ_0069523	0.009687525	-1.314486744
hsa_circ_0021372	0.009700034	-1.263448154
hsa_circ_0008777	0.00970108	-2.114948102
hsa_circ_0016640	0.00970108	-1.545920612
hsa_circ_0016119	0.00970108	-1.301333835
hsa_circ_0045020	0.009716396	-2.992285865
hsa_circ_0095649	0.009716396	-2.464732033
hsa_circ_0070986	0.009716396	-1.730793793
hsa_circ_0002975	0.009716396	-1.667257853

hsa_circ_0120852	0.009716396	-1.191518184
hsa_circ_0070032	0.009716396	-1.045373764
hsa_circ_0035655	0.009726219	-1.304390946
hsa_circ_0125792	0.009762649	-2.365886383
hsa_circ_0033132	0.009762649	-2.066819079
hsa_circ_0071683	0.009762649	-1.836771843
hsa-circRNA11180-32	0.009762649	-1.202055495
hsa_circ_0081418	0.009774297	-1.710040349
hsa_circ_0086373	0.009777536	-1.770723288
hsa_circ_0136858	0.009794694	-2.841137114
hsa_circ_0124784	0.009794694	-1.137759686
hsa_circ_0003133	0.009901196	-1.218001046
hsa_circ_0075001	0.009907828	-1.341047189
hsa_circ_0016557	0.009946502	-1.245620558
hsa_circ_0082457	0.009949346	-1.308792934
hsa_circ_0086371	0.009956307	-1.931320373
hsa_circ_0116783	0.009956307	-1.448876094
hsa_circ_0023982	0.009971684	-1.584447741
hsa_circ_0118495	0.009971684	-1.129912525
hsa_circ_0068041	0.009971684	-1.033137902
