# Supplementary material for: Integrative Analysis Extracts a Core ceRNA Network of the Fetal Hippocampus With Down Syndrome
Source: Front Genet. 2020 Nov 30;11:565955. doi: 10.3389/fgene.2020.565955 (PMC7735064; doi:10.3389/fgene.2020.565955)
Supplement: Supplementary Table 3 — The expression of genes shown in Figure 8A. [file Table_3.PDF]

Supplementary Table 3. The expression of genes shown in Figure 7A.

Gene	ModuleColor	Log2FC	q-value
SRI	yellow	1.792689	0
GPM6B	yellow	1.93693	0
PON2	yellow	1.659422	0.001461
PSAT1	yellow	1.712684	0.001461
OLIG1	yellow	2.435095	0.003772
LUZP2	yellow	2.904041	0.004089
HAPLN1	yellow	3.593832	0.004089
PMP2	yellow	2.482668	0.006163
SIRT2	yellow	1.56306	0.007942
HEPN1	yellow	2.175205	0.007942
KCNIP1	yellow	2.360505	0.007942
RAMP1	yellow	2.388713	0.007942
FIBIN	yellow	2.474436	0.007942
ALCAM	yellow	2.641777	0.007942
PPAP2B	yellow	1.458959	0.008051
TRIB2	yellow	1.111432	0.008275
SERPINE2	yellow	2.182946	0.008997
PCDH17	yellow	1.938173	0.010898
FGFBP3	yellow	-1.22567	0.011884
PLEKHB1	yellow	2.672538	0.011884
NT5E	yellow	2.798569	0.011884
EDNRB	yellow	3.497625	0.011884
SCRG1	yellow	0.885731	0.012558
RGCC	yellow	1.701283	0.013025
CMTM5	yellow	0.793771	0.013965
MT3	yellow	2.067191	0.013965
LHFPL3	yellow	1.882917	0.015021
RAB31	yellow	2.423605	0.015306
PTPRZ1	yellow	0.768417	0.015972
KANK1	yellow	1.455492	0.016864
TRAF4	yellow	-0.52886	0.019097
CSPG5	yellow	1.861757	0.019706
CADM2	yellow	2.257252	0.020243
BRINP1	yellow	1.699818	0.020881
BCAN	yellow	1.079293	0.022954
GPR17	yellow	2.902884	0.022954
SLC44A1	yellow	-0.85831	0.028699
CNTN1	yellow	2.840866	0.028699
PDGFRA	yellow	2.085561	0.031749
LPPR1	yellow	1.717517	0.033275

CNP	yellow	0.773659	0.034691
S100B	yellow	2.718395	0.034691
NTRK2	yellow	1.487126	0.036649
PDE4B	yellow	1.20201	0.03889
LRRC4C	yellow	1.96997	0.03889
TMEM100	yellow	1.840806	0.041114
APOD	yellow	1.349309	0.043534
OLIG2	yellow	1.380563	0.046327
METRN	yellow	0.758559	0.050437
ASIC4	yellow	0.8118	0.052556
SHISA4	yellow	0.814509	0.055022
DBI	yellow	0.779386	0.057935
TTYH1	yellow	0.612494	0.059773
NKAIN4	yellow	0.949722	0.059773
DNER	yellow	0.766383	0.076471
PDLIM5	yellow	0.714136	0.078939
TMOD1	yellow	1.11836	0.082095
C2orf80	yellow	1.399827	0.082095
CHRM3	yellow	2.033405	0.082095
C1QL1	yellow	0.440421	0.089271
COL20A1	yellow	0.799087	0.089271
ASCL1	yellow	-1.27509	0.104265
BRINP3	yellow	0.792106	0.108002
ITM2A	yellow	0.801573	0.111523
UGDH	yellow	-0.37632	0.115469
SOX6	yellow	-0.64792	0.122193
SNX22	yellow	0.787349	0.122193
CA10	yellow	1.861082	0.12624
SLITRK2	yellow	0.480162	0.137447
TMSB4X	yellow	-0.26534	0.1609
PPAPDC1A	yellow	1.167229	0.17386
OPCML	yellow	1.052277	0.193198
COL9A3	yellow	-0.59009	0.198569
C8orf46	yellow	1.24811	0.198569
C1orf61	yellow	0.223423	0.205489
OLFM2	yellow	0.591296	0.212721
PROM1	yellow	-0.57949	0.226356
GRB14	yellow	-0.33606	0.226356
HEY1	yellow	0.326537	0.239369
CTHRC1	yellow	0.715981	0.239369
PHGDH	yellow	0.283211	0.246118
NCALD	yellow	0.819014	0.246118

LAPTM4B	yellow	0.380508	0.253529
ETV1	yellow	0.474981	0.261428
ARL4A	yellow	0.403486	0.302781
SOX8	yellow	0.654802	0.302781
TNK2	yellow	0.308361	0.315956
BAMBI	yellow	0.606726	0.323056
SMOC1	yellow	-0.13195	0.349095
NXPH1	yellow	0.469573	0.354794
MMP16	yellow	-0.12438	0.371256
DLL3	yellow	0.228234	0.382444
FABP7	yellow	-0.08421	0.388511
PCDH9	yellow	-0.13876	0.42952
SCD5	yellow	0.082975	0.42952
TCF7L2	yellow	0.511569	0.437177
SOX2	yellow	-0.11216	0.444065
LIMA1	yellow	0.057416	0.480768
NOVA1	yellow	0.031677	0.487402
SLC9A9	turquoise	1.265857	0.001461
P2RY12	turquoise	2.488129	0.001461
GATM	turquoise	2.840765	0.001461
BST2	turquoise	3.324724	0.001461
SIRPA	turquoise	2.188717	0.003772
CSF1R	turquoise	1.07779	0.004089
APOE	turquoise	3.166362	0.004089
GLUL	turquoise	1.210389	0.006163
ASAH1	turquoise	1.786262	0.006163
IFRD1	turquoise	0.815493	0.007942
CTSB	turquoise	1.110965	0.007942
MEF2C	turquoise	1.534062	0.007942
GPR34	turquoise	1.960919	0.007942
CX3CR1	turquoise	2.382999	0.007942
SKAP2	turquoise	2.938211	0.007942
RNASET2	turquoise	0.753262	0.008051
DPP7	turquoise	1.166008	0.008051
C3	turquoise	2.295664	0.008051
IFNGR1	turquoise	1.010135	0.008997
PPT1	turquoise	1.037734	0.008997
ITPR2	turquoise	1.772308	0.008997
ARPC1B	turquoise	0.889551	0.009863
XBP1	turquoise	1.115832	0.011884
HIST1H2BK	turquoise	1.154065	0.012558
CREG1	turquoise	1.460323	0.012558

LGMN	turquoise	1.169348	0.012963
LAPTM5	turquoise	1.424815	0.015306
BHLHE41	turquoise	2.741014	0.015972
SCG3	turquoise	0.954196	0.016864
APBB1IP	turquoise	1.785969	0.016864
SERPINB9	turquoise	1.927252	0.016864
SAMSN1	turquoise	2.449297	0.01788
ITM2B	turquoise	1.00137	0.019706
FOS	turquoise	2.331849	0.019706
CHCHD10	turquoise	1.123136	0.020243
IL13RA1	turquoise	1.227926	0.021791
C10orf54	turquoise	1.032947	0.022954
GNG7	turquoise	2.56718	0.022954
FTL	turquoise	0.359634	0.023325
FTH1	turquoise	0.801904	0.023325
EGR2	turquoise	2.118526	0.023325
ABCG2	turquoise	0.965987	0.024261
HERPUD1	turquoise	0.843502	0.024763
TLR4	turquoise	1.227987	0.024763
HTRA1	turquoise	1.468896	0.027412
EGR3	turquoise	2.502382	0.027412
PFN1	turquoise	-0.5632	0.028699
GLIPR1	turquoise	1.413161	0.028699
OLR1	turquoise	1.752749	0.028699
UCP2	turquoise	-1.27195	0.030268
KCTD12	turquoise	1.219958	0.030268
CXCL16	turquoise	0.717561	0.033275
FYB	turquoise	1.865206	0.034691
C1QC	turquoise	1.14646	0.036649
OSTF1	turquoise	1.337882	0.036649
HIST1H1C	turquoise	-1.04454	0.03889
PLA2G16	turquoise	1.272083	0.03889
IGF1	turquoise	1.590051	0.03889
APOC1	turquoise	1.413811	0.041114
RGS10	turquoise	1.757749	0.041114
RGS19	turquoise	0.93463	0.046327
SLCO2B1	turquoise	1.251628	0.046327
CTSH	turquoise	2.13852	0.050437
LST1	turquoise	1.495235	0.052556
MGAT4A	turquoise	1.561888	0.052556
IPCEF1	turquoise	1.697818	0.055022
CD300A	turquoise	0.813443	0.057935

SORL1	turquoise	1.345851	0.057935
ETS2	turquoise	1.393471	0.057935
A2M	turquoise	1.155684	0.062241
NAA20	turquoise	0.419755	0.066096
IER2	turquoise	0.416948	0.070083
LHFPL2	turquoise	1.03083	0.070083
LCP1	turquoise	1.337597	0.070083
SMYD3	turquoise	0.548239	0.072253
GADD45B	turquoise	1.583134	0.072253
RHOB	turquoise	1.061845	0.076471
MDK	turquoise	-1.10656	0.078939
BIN1	turquoise	0.652142	0.082095
TRIB1	turquoise	0.904657	0.085713
TPP1	turquoise	0.413052	0.089271
LIPA	turquoise	0.601697	0.089271
C12orf75	turquoise	0.677621	0.089271
CD83	turquoise	0.920293	0.089271
SGK1	turquoise	1.479127	0.089271
B3GNT5	turquoise	-1.05409	0.092381
TYROBP	turquoise	0.634222	0.096514
ARRB2	turquoise	-0.40794	0.099866
ENTPD1	turquoise	0.653885	0.099866
TREM2	turquoise	1.044604	0.099866
C1QB	turquoise	0.874128	0.104265
RGS2	turquoise	1.541317	0.104265
PARVG	turquoise	-0.19281	0.108002
SLC7A8	turquoise	0.656542	0.108002
FOLR2	turquoise	1.06371	0.111523
PPP1R15A	turquoise	-0.24537	0.115469
HIST1H2AC	turquoise	0.635244	0.115469
ARHGDIB	turquoise	0.82985	0.115469
VSIG4	turquoise	1.152313	0.115469
MGST2	turquoise	0.69635	0.118529
PDK4	turquoise	0.813525	0.118529
CPE	turquoise	1.037242	0.118529
FCGR3A	turquoise	1.396708	0.118529
AP1B1	turquoise	0.322851	0.122193
HSD17B14	turquoise	0.495797	0.122193
SYNGR2	turquoise	0.566182	0.122193
PIK3IP1	turquoise	0.741402	0.122193
FABP5	turquoise	-0.45206	0.12624
BAG3	turquoise	0.913876	0.12624

NR4A2	turquoise	-1.33752	0.129867
DNAJB1	turquoise	0.294782	0.129867
SLC29A3	turquoise	0.367483	0.129867
FUOM	turquoise	-0.5473	0.134018
POLD4	turquoise	0.272143	0.137447
ZFP36	turquoise	0.931532	0.137447
CEBPD	turquoise	1.119688	0.141143
PLXDC2	turquoise	0.528771	0.151023
ZNF467	turquoise	0.961475	0.151023
CPVL	turquoise	1.13014	0.155651
NAGA	turquoise	-0.32717	0.166709
JUNB	turquoise	0.375623	0.166709
HSD17B11	turquoise	-0.36978	0.17386
TMC6	turquoise	-0.1602	0.17386
CSRNP1	turquoise	0.258941	0.17386
GSN	turquoise	0.808426	0.17386
RBM47	turquoise	-1.42895	0.179326
AIF1	turquoise	0.781066	0.186663
GPNMB	turquoise	1.239581	0.186663
ARID5A	turquoise	-0.17218	0.193198
NFIA	turquoise	-0.74492	0.198569
SAT1	turquoise	0.540523	0.198569
LPAR6	turquoise	0.73786	0.198569
IER3	turquoise	-0.59902	0.205489
DUSP1	turquoise	0.204391	0.226356
PTPRE	turquoise	0.99834	0.226356
SOCS6	turquoise	0.306029	0.232731
EHD4	turquoise	0.516923	0.232731
ARHGAP4	turquoise	-0.13068	0.239369
CNPY3	turquoise	-0.12658	0.239369
CTSL	turquoise	0.339822	0.239369
NINJ1	turquoise	0.246712	0.246118
DNASE2	turquoise	0.361881	0.246118
SMAP2	turquoise	0.370388	0.246118
S100A11	turquoise	0.736388	0.246118
CAPG	turquoise	-0.28297	0.261428
PLSCR1	turquoise	0.260146	0.261428
CORO1A	turquoise	0.362666	0.261428
B4GALT1	turquoise	-0.28156	0.270132
CHCHD7	turquoise	-0.26188	0.270132
SRGN	turquoise	0.669662	0.285797
NR4A1	turquoise	0.253747	0.294608

QPRT	turquoise	0.258941	0.294608
CNN3	turquoise	0.32987	0.294608
NUDT14	turquoise	0.219587	0.302781
SPP1	turquoise	0.840363	0.302781
GYPC	turquoise	-0.18623	0.309545
ACTG1	turquoise	-0.18426	0.309545
CD74	turquoise	0.151339	0.309545
PDPN	turquoise	-0.27056	0.315956
RHOG	turquoise	0.124063	0.315956
CTSD	turquoise	0.200128	0.315956
OLFML3	turquoise	0.533065	0.315956
SERPINF1	turquoise	-0.66406	0.323056
TXN	turquoise	-0.13622	0.323056
RNASE1	turquoise	0.36793	0.323056
GRIA2	turquoise	0.593688	0.323056
MCL1	turquoise	-0.09265	0.342685
GPX1	turquoise	0.168385	0.342685
RIN2	turquoise	0.352194	0.342685
KLF2	turquoise	0.218967	0.349095
FCGRT	turquoise	0.280125	0.349095
IFI16	turquoise	0.433066	0.349095
TM6SF1	turquoise	0.224534	0.354794
ZFP36L2	turquoise	0.228111	0.354794
GLRX	turquoise	-0.26986	0.36068
LGALS9	turquoise	-0.06733	0.36068
ZFHX3	turquoise	0.735522	0.36068
LYN	turquoise	0.697952	0.366188
GAS6	turquoise	0.210514	0.371256
PFN2	turquoise	-0.13971	0.382444
RAB32	turquoise	-0.12438	0.382444
MAF	turquoise	0.229588	0.382444
DHRS3	turquoise	-0.22415	0.388511
OTUD1	turquoise	0.165044	0.388511
MARCKSL1	turquoise	-0.07552	0.393692
PHACTR1	turquoise	0.31429	0.393692
ZFP36L1	turquoise	0.133432	0.402921
CD14	turquoise	0.507008	0.407996
JUN	turquoise	0.19232	0.413227
CYBA	turquoise	0.177854	0.417401
RASSF4	turquoise	0.071488	0.425379
STAB1	turquoise	0.106884	0.437177
BLVRB	turquoise	0.179638	0.437177

IRF5	turquoise	0.035061	0.447631
CTSC	turquoise	-0.08758	0.450967
GAL3ST4	turquoise	0.085697	0.454476
COTL1	turquoise	0.235727	0.46063
NFKBIA	turquoise	0.070389	0.47101
MFSD1	turquoise	-0.02107	0.477617
NPC2	turquoise	0.073546	0.477617
KLF6	turquoise	0.073135	0.481557
MAFB	turquoise	0.042644	0.490278
NFATC2	turquoise	0.053667	0.49108
BTG2	turquoise	0.01035	0.492375
CDKN1A	turquoise	0.011066	0.492375
GRN	turquoise	0.017209	0.492375
FCER1G	turquoise	0.034075	0.492375
GPC2	red	-1.88577	0.005264
CST3	red	2.327141	0.007942
SOX11	red	-1.55512	0.008525
TUBB	red	-1.0807	0.008637
SNAP25	red	2.970412	0.012558
NFIB	red	-1.38685	0.015306
NNAT	red	-1.09634	0.015972
B2M	red	1.250962	0.024261
STMN1	red	-0.64363	0.031749
DRAXIN	red	-0.86355	0.036649
ELAVL4	red	-1.14944	0.041114
TUBB2B	red	-0.26015	0.050437
ENO2	red	0.774249	0.050437
KLHL35	red	-0.83367	0.055022
ELAVL3	red	-0.42954	0.062241
SLC1A3	red	1.043345	0.062241
PCSK1N	red	1.386314	0.062241
HN1	red	-0.33388	0.066096
BCL11A	red	-0.85387	0.072253
TUBB2A	red	0.305562	0.072253
SYT1	red	0.998123	0.078939
GNG3	red	1.406047	0.082095
OCIAD2	red	-0.51891	0.089271
KLC1	red	0.54666	0.096514
KIF5C	red	0.774502	0.096514
RTN1	red	0.514905	0.104265
ATP6V1G2	red	1.52672	0.115469
THRA	red	0.567059	0.134018

NEUROD2	red	-2.07157	0.151023
GNG5	red	-0.44851	0.155651
TMSB10	red	-0.18492	0.155651
ANXA5	red	0.475915	0.166709
SEZ6L2	red	0.585539	0.166709
LY6H	red	0.843662	0.166709
RAB3A	red	0.786513	0.17386
CRMP1	red	-0.3357	0.179326
GAP43	red	0.700617	0.186663
PPP2R2B	red	1.362217	0.186663
NEUROD6	red	-1.63397	0.198569
CXADR	red	0.508023	0.198569
MAPT	red	0.571337	0.226356
SH3BP5	red	-0.34887	0.232731
STMN4	red	0.630871	0.232731
CAMKV	red	1.099026	0.253529
ENC1	red	-0.59488	0.261428
BEX2	red	0.4654	0.270132
UCHL1	red	0.263275	0.277436
MLLT11	red	-0.13289	0.285797
PODXL2	red	0.310805	0.323056
APLP1	red	0.297015	0.3289
BHLHE22	red	-0.92906	0.388511
BASP1	red	0.07902	0.413227
MAP1B	red	0.097206	0.413227
HSPB1	red	-0.08835	0.440945
TUBA1A	red	0.048934	0.447631
NSG1	red	0.076559	0.477617
STMN2	red	0.026871	0.485463
FAM167A	green	1.974603	0.007942
PCP4	green	2.860069	0.008997
FRRS1L	green	1.934894	0.01788
C11orf87	green	2.687845	0.021791
KLHL13	green	1.921132	0.025835
CACNA2D3	green	2.238451	0.027412
ISOC1	green	0.693855	0.030268
B3GNT4	green	1.013927	0.034691
NDRG4	green	1.498047	0.046327
FAT3	green	1.720278	0.050437
ARHGDIG	green	1.491237	0.052556
IGFBP5	green	1.613579	0.062241
PPAP2C	green	0.941257	0.072253

PHLDA3	green	0.692516	0.076471
QPCT	green	1.654115	0.078939
WFIKKN2	green	-2.12281	0.082095
CYGB	green	0.5065	0.085713
SYNGR3	green	2.047329	0.089271
CDKN2D	green	0.886433	0.092381
FSTL5	green	-0.45383	0.108002
GNAL	green	2.018029	0.108002
AMIGO2	green	1.340676	0.111523
RALYL	green	1.566182	0.111523
STX1A	green	1.101852	0.115469
DUSP23	green	0.932061	0.118529
CNTNAP2	green	1.269512	0.12624
ST6GALNAC5	green	1.031536	0.137447
CALB1	green	1.690775	0.145694
ABLIM1	green	1.271127	0.151023
SHISA2	green	-1.25739	0.155651
DMTN	green	0.627887	0.193198
TRH	green	1.157561	0.212721
NHLH2	green	-1.38082	0.246118
ZNF503	green	0.197865	0.270132
C1QTNF3	green	-0.57669	0.285797
CALB2	green	0.605305	0.285797
FBLN2	green	0.172744	0.302781
CLUL1	green	0.565012	0.309545
FAM155A	green	0.772308	0.309545
GREM2	green	0.834954	0.335468
C7	green	-0.44399	0.349095
CHST8	green	-0.49859	0.36068
RELN	green	0.766892	0.382444
ZIC2	green	-0.37407	0.417401
TPBG	green	-0.23786	0.417401
EBF3	green	-0.02547	0.444065
RSPO3	green	-0.35458	0.457499
CCK	green	-0.20124	0.46063
SLC35F2	green	-0.09696	0.46542
RSPO2	green	-0.12658	0.475429
NPB	green	-0.00796	0.478785
TAGLN2	green	-0.00087	0.478785
CASC5	brown	-2.6144	0.005264
TTK	brown	-2.92358	0.008525
NCAPG	brown	-2.67669	0.008525

CCNA2	brown	-2.5454	0.008525
CENPN	brown	-2.50964	0.008525
MKI67	brown	-2.26604	0.008525
EZH2	brown	-3.05529	0.008555
NUF2	brown	-2.95009	0.008555
CENPF	brown	-2.773	0.008555
CKS2	brown	-2.0619	0.008555
CENPW	brown	-1.87039	0.008555
DLGAP5	brown	-2.99885	0.008637
NUSAP1	brown	-2.72166	0.008637
DTL	brown	-2.47875	0.008637
KIAA0101	brown	-2.37034	0.008637
SMC2	brown	-1.28279	0.008637
UBE2C	brown	-2.66841	0.008997
AURKB	brown	-2.54288	0.008997
CCNB2	brown	-2.66658	0.009863
KIF15	brown	-2.61794	0.009863
CDCA3	brown	-2.30757	0.010898
AURKA	brown	-2.00173	0.011884
NDC80	brown	-2.62238	0.012694
CDC20	brown	-2.56064	0.012694
BIRC5	brown	-2.38458	0.012694
GINS2	brown	-2.20823	0.012694
OIP5	brown	-1.96184	0.012694
DEPDC1B	brown	-2.82317	0.012963
SPC24	brown	-2.20091	0.012963
HMGB2	brown	-2.31905	0.013965
TMPO	brown	-2.17984	0.013965
CENPH	brown	-1.46395	0.013965
SPC25	brown	-2.69152	0.015021
MCM10	brown	-2.1078	0.015306
SGOL2	brown	-2.00058	0.015306
TACC3	brown	-1.92467	0.015306
FANCI	brown	-1.85778	0.015306
RNASEH2A	brown	-1.38421	0.015306
HMMR	brown	-1.68223	0.015972
KIF11	brown	-2.52284	0.016864
PRC1	brown	-2.21023	0.016864
CDCA5	brown	-2.26743	0.01788
RTKN2	brown	-1.35771	0.01788
BUB3	brown	-1.03446	0.019097
FAM64A	brown	-2.47313	0.019706

SPAG5	brown	-2.2109	0.019706
KIF2C	brown	-2.15457	0.019706
BARD1	brown	-1.56961	0.019706
FAM111A	brown	-1.55512	0.019706
CCNF	brown	-1.21591	0.020881
PHF19	brown	-1.12155	0.020881
ESCO2	brown	-2.25981	0.021791
HELLS	brown	-2.17984	0.021791
H2AFZ	brown	-1.00781	0.021791
ASPM	brown	-2.45403	0.022954
CDCA4	brown	-1.31905	0.022954
CENPU	brown	-2.67946	0.023325
RRM2	brown	-2.30401	0.023325
TPX2	brown	-2.13924	0.023325
NEK2	brown	-2.08498	0.023325
DBF4	brown	-1.71217	0.023325
FBXO5	brown	-1.97937	0.024261
H2AFV	brown	-1.23684	0.024261
MXD3	brown	-1.62816	0.025835
PBK	brown	-2.21759	0.027412
CENPE	brown	-1.60735	0.027412
H2AFX	brown	-1.14944	0.027412
DSN1	brown	-1.17951	0.030268
RAN	brown	-1.16618	0.030268
TYMS	brown	-2.05649	0.031749
CDKN3	brown	-1.49248	0.031749
CKS1B	brown	-1.33352	0.031749
KNSTRN	brown	-1.27439	0.031749
LMNB2	brown	-1.19067	0.031749
CENPM	brown	-1.52036	0.033275
RFC3	brown	-0.63129	0.034691
MIS18BP1	brown	-1.68594	0.034691
NCAPD2	brown	-1.65199	0.034691
KIFC1	brown	-2.02621	0.036649
CENPK	brown	-1.95736	0.036649
PCNA	brown	-1.32229	0.036649
UBE2S	brown	-0.84684	0.03889
NUCKS1	brown	-0.3681	0.03889
NMU	brown	-1.50471	0.03889
DEK	brown	-1.12469	0.03889
HMGB1	brown	-0.63308	0.041114
CCNB1	brown	-1.58944	0.041114

CHEK1	brown	-1.48965	0.041114
RAD21	brown	-0.43245	0.043534
ANLN	brown	-1.55554	0.046327
TOP2A	brown	-2.23718	0.049316
CDK1	brown	-1.90401	0.049316
SMC4	brown	-1.8625	0.049316
MAD2L1	brown	-1.77794	0.049316
ATAD2	brown	-1.30401	0.049316
ECT2	brown	-1.32771	0.050437
GMNN	brown	-1.20457	0.050437
UBE2T	brown	-0.90942	0.050437
PKMYT1	brown	-0.51643	0.052556
KIF22	brown	-1.10067	0.055022
LSM5	brown	-0.84451	0.055022
ANP32E	brown	-1.12784	0.057935
RACGAP1	brown	-1.27928	0.059773
CDKN2C	brown	-1.51169	0.062241
BUB1B	brown	-1.88683	0.066096
ZWINT	brown	-1.39327	0.066096
ORC6	brown	-1.08896	0.066096
DTYMK	brown	-0.88337	0.066096
NDE1	brown	-0.74178	0.066096
PSRC1	brown	-0.94092	0.070083
TRIP13	brown	-0.93374	0.072253
DDX39A	brown	-0.95093	0.076471
HMGB3	brown	-0.88657	0.076471
CKAP2	brown	-0.85022	0.078939
RPA3	brown	-0.55788	0.078939
ARL6IP1	brown	1.644641	0.082095
DHFR	brown	-0.76857	0.085713
BTG3	brown	-0.51808	0.108002
FEN1	brown	-0.73504	0.111523
TK1	brown	-0.71076	0.111523
HMGN2	brown	-0.52492	0.115469
CCDC34	brown	-0.58814	0.12624
CDC25B	brown	-0.80213	0.134018
EMC9	brown	-0.22871	0.179326
SAPCD2	brown	-0.62905	0.198569
KIAA1524	brown	-0.47333	0.198569
MNS1	brown	-0.90969	0.205489
WDR34	brown	-0.19182	0.212721
SFRP2	brown	-0.95512	0.261428

MZT1	brown	-0.1565	0.294608
GPSM2	brown	-0.27649	0.342685
CCNA1	brown	0.230695	0.36068
DCXR	brown	0.18053	0.37675
DIAPH3	brown	-0.1665	0.393692
GGH	brown	-0.08727	0.42952
APOLD1	brown	0.178492	0.437177
TUBB4B	brown	0.124725	0.46063
TUBA1B	brown	0.051163	0.46286
APLNR	blue	2.79221	0
F3	blue	3.613437	0
KCNJ8	blue	1.255561	0.001461
ISG15	blue	1.545029	0.001461
RASL12	blue	2.019631	0.001461
ITM2C	blue	3.239138	0.001461
MYLK	blue	2.814796	0.002703
RASGRP2	blue	2.9164	0.003772
SLC40A1	blue	1.140844	0.004089
ARHGAP26	blue	2.904927	0.004089
SPATS2L	blue	1.088074	0.006163
TNS3	blue	1.121679	0.007942
SPARC	blue	1.279947	0.007942
PLXDC1	blue	1.356313	0.007942
ADORA2B	blue	1.447791	0.007942
PRRX1	blue	1.593115	0.007942
PMP22	blue	1.960808	0.007942
ABCC9	blue	2.082839	0.007942
SPARCL1	blue	3.68245	0.007942
AFAP1L2	blue	1.334225	0.008051
RGS5	blue	2.024674	0.008051
CYTH3	blue	1.049631	0.008275
ATP1A2	blue	1.419755	0.008432
TIMP3	blue	1.448002	0.008432
SEMA5A	blue	1.182565	0.011884
GUCY1B3	blue	2.341189	0.011884
PDZD2	blue	2.63143	0.011884
GUCY1A3	blue	3.422448	0.011884
MGLL	blue	2.449588	0.013025
SMOC2	blue	2.740496	0.013965
FN1	blue	1.268375	0.016864
CCDC3	blue	1.548141	0.01788
FERMT2	blue	1.059009	0.019097

TJP1	blue	0.682304	0.020243
SYNM	blue	1.980976	0.020881
SMTN	blue	-0.53491	0.022954
SLC6A1	blue	1.302933	0.022954
ITGB1	blue	-0.89727	0.023325
ANXA6	blue	0.883855	0.024261
MT2A	blue	1.066744	0.024261
HIGD1B	blue	1.251871	0.025835
CD9	blue	1.87342	0.025835
TPM2	blue	-1.02826	0.027412
TESC	blue	2.038682	0.027412
KANK3	blue	0.575119	0.028699
DLC1	blue	1.743127	0.028699
DAAM2	blue	2.536103	0.031749
RRAS	blue	0.451963	0.033275
TSPAN12	blue	0.994652	0.033275
SYTL2	blue	1.245252	0.033275
GPR116	blue	1.264777	0.033275
DMD	blue	-0.92057	0.03889
CDH11	blue	0.768163	0.041114
STOM	blue	1.028923	0.041114
TGFBI	blue	1.89103	0.043534
NR2F2	blue	-1.32988	0.049316
PTN	blue	1.035342	0.049316
MDFI	blue	0.852079	0.050437
LZTS1	blue	-1.13353	0.055022
TPM4	blue	-0.63285	0.057935
COL9A1	blue	-1.56362	0.059773
HES1	blue	-0.73889	0.059773
SLC9A1	blue	0.384823	0.059773
COL4A1	blue	1.060532	0.059773
B3GNT2	blue	1.982181	0.059773
ARHGEF17	blue	0.429429	0.062241
AXL	blue	0.595599	0.062241
PRELP	blue	0.784253	0.066096
ST3GAL5	blue	1.165494	0.066096
ITIH5	blue	1.197425	0.066096
IL34	blue	0.900645	0.070083
ID3	blue	1.019417	0.070083
COL1A2	blue	-1.67761	0.072253
COX7A1	blue	0.558072	0.072253
EPAS1	blue	1.185613	0.072253

PRKCB	blue	1.866077	0.072253
CLIC1	blue	-0.83393	0.076471
SGCE	blue	0.587269	0.076471
SPRY1	blue	0.854315	0.076471
PDE8B	blue	1.175301	0.076471
GRM3	blue	1.9775	0.076471
GJC1	blue	-0.75098	0.078939
MAP1LC3A	blue	1.119489	0.078939
ECE1	blue	0.24987	0.082095
GABRD	blue	0.910886	0.082095
DYNLT3	blue	1.114234	0.082095
TNFRSF21	blue	1.16395	0.085713
SIGIRR	blue	-0.8396	0.089271
THY1	blue	1.437281	0.089271
GGT5	blue	0.333996	0.092381
RAPGEF5	blue	1.42605	0.096514
ETS1	blue	0.505078	0.104265
LHFP	blue	0.898866	0.104265
CKB	blue	-0.46335	0.108002
ESAM	blue	0.694479	0.108002
RBMS1	blue	1.004897	0.111523
MYL9	blue	-0.32139	0.115469
EDNRA	blue	1.279115	0.115469
IQGAP1	blue	-0.60823	0.118529
FILIP1	blue	0.891575	0.118529
PELO	blue	0.255199	0.122193
EGFL7	blue	0.640528	0.122193
PMEPA1	blue	1.107621	0.122193
LMNA	blue	-0.43596	0.12624
CD99	blue	0.789938	0.12624
COL6A1	blue	0.614851	0.129867
ITGB5	blue	0.621524	0.129867
HSPA2	blue	0.788352	0.129867
TGFB1I1	blue	-0.31941	0.134018
SIK1	blue	0.435949	0.134018
C1orf54	blue	0.626299	0.134018
GNG11	blue	0.635151	0.134018
CAV2	blue	1.255319	0.134018
PALD1	blue	0.423202	0.137447
CPQ	blue	0.526069	0.137447
TGFBR2	blue	0.928958	0.137447
NDUFA4	blue	0.292664	0.141143

FOXQ1	blue	0.858299	0.141143
SVIL	blue	-0.81401	0.145694
FOXF2	blue	0.614097	0.145694
PAPSS2	blue	0.618145	0.145694
SLC20A2	blue	0.699196	0.145694
LAMA4	blue	0.337768	0.151023
GALNT18	blue	0.928806	0.151023
WLS	blue	0.875544	0.155651
CHST2	blue	1.302348	0.155651
VIM	blue	-0.53345	0.1609
SELM	blue	1.012926	0.1609
PDLIM1	blue	-0.662	0.166709
FKBP5	blue	-0.53011	0.17386
PLOD1	blue	0.264476	0.17386
RBMS3	blue	0.899408	0.17386
SERPING1	blue	-0.53178	0.179326
TBXA2R	blue	-0.20923	0.179326
RHOC	blue	0.468218	0.179326
COL4A2	blue	0.557582	0.179326
ENG	blue	0.393965	0.186663
LAMB2	blue	-0.31815	0.193198
IFITM1	blue	1.096734	0.193198
FRZB	blue	0.367259	0.205489
CA2	blue	0.412185	0.205489
GPRC5C	blue	0.646163	0.205489
VAMP5	blue	0.803144	0.205489
TFPI	blue	-0.34081	0.212721
TUBB6	blue	-0.35734	0.226356
NXPH4	blue	-0.10718	0.226356
TMEM88	blue	-0.34282	0.246118
SYDE1	blue	-0.18606	0.246118
LAMC1	blue	0.412185	0.246118
ARHGAP29	blue	0.696884	0.246118
COL1A1	blue	-0.42391	0.253529
MYH9	blue	0.22367	0.253529
COL6A2	blue	0.450697	0.253529
CAV1	blue	0.669208	0.253529
C11orf96	blue	-0.45245	0.261428
PHLDB2	blue	-0.33461	0.261428
ISYNA1	blue	-0.25498	0.270132
C16orf80	blue	-0.17886	0.270132
EBF1	blue	1.000649	0.270132

TLE1	blue	-0.19034	0.277436
EPS8	blue	0.309176	0.277436
SH2D3C	blue	0.359296	0.277436
IFITM3	blue	0.449535	0.277436
SLC9A3R1	blue	0.228726	0.285797
GPR124	blue	0.437973	0.285797
ABCA8	blue	-0.95344	0.294608
DCN	blue	0.88096	0.294608
MSX1	blue	-0.65381	0.302781
ANXA2	blue	-0.53199	0.302781
MYO1B	blue	-0.40125	0.302781
PARVA	blue	0.285816	0.309545
CALD1	blue	-0.30561	0.315956
AKAP12	blue	0.317999	0.315956
MFGE8	blue	-0.10936	0.323056
COL21A1	blue	-0.52056	0.335468
UACA	blue	-0.31151	0.335468
PLTP	blue	0.413702	0.335468
COL3A1	blue	-0.67208	0.342685
CLEC11A	blue	0.120352	0.342685
VASN	blue	0.216238	0.342685
TMEM109	blue	0.251931	0.342685
NID1	blue	0.401303	0.342685
ACTA2	blue	0.80554	0.342685
SLC12A2	blue	0.229588	0.349095
SERPINH1	blue	0.28238	0.349095
LPL	blue	0.848398	0.354794
PDGFRB	blue	-0.16408	0.36068
EFEMP2	blue	0.141825	0.366188
IFITM2	blue	0.334568	0.366188
COBLL1	blue	0.359746	0.366188
ANXA1	blue	0.463309	0.366188
KLHDC8B	blue	0.258579	0.371256
LAMB1	blue	-0.35071	0.37675
ZNF703	blue	-0.15136	0.37675
BGN	blue	0.139993	0.382444
CTGF	blue	0.311736	0.388511
PDLIM2	blue	-0.10129	0.393692
S1PR3	blue	-0.21474	0.397969
PCOLCE	blue	0.066399	0.407996
ADAMTS1	blue	0.389126	0.407996
LGALS3BP	blue	-0.11029	0.421087

ACTN1	blue	0.223917	0.421087
COL5A2	blue	0.137372	0.425379
LGALS1	blue	-0.10594	0.42952
CD63	blue	0.129876	0.42952
PRSS23	blue	0.207768	0.42952
MGP	blue	0.461633	0.42952
FSTL1	blue	-0.09712	0.433665
CD248	blue	-0.09604	0.433665
AK1	blue	0.242328	0.433665
VCL	blue	0.178619	0.437177
STOML3	blue	-0.17641	0.440945
RFTN1	blue	-0.11982	0.440945
HEYL	blue	-0.03549	0.440945
GMDS	blue	0.125651	0.440945
TAGLN	blue	0.223175	0.440945
SNRK	blue	-0.05889	0.444065
KANK2	blue	-0.07994	0.450967
MYL12A	blue	0.110363	0.450967
AGRN	blue	-0.04008	0.454476
LINGO1	blue	0.165044	0.454476
CRIP1	blue	0.179001	0.454476
NOTCH3	blue	-0.01304	0.46542
MRC2	blue	-0.01888	0.468416
EMP2	blue	0.085561	0.473038
RFXANK	blue	-0.0113	0.477617
COL18A1	blue	-0.04246	0.478785
SLC12A7	blue	-0.0068	0.478785
GPER1	blue	-0.00549	0.478785
ECSCR	blue	-0.00072	0.478785
FOXC1	blue	0.121679	0.481557
COLEC12	blue	0.12075	0.482529
IGFBP7	blue	0.075464	0.484471
CTSK	blue	0.076149	0.484471
SLC6A13	blue	0.02432	0.486186
LAMA2	blue	0.080794	0.487402
SEPP1	blue	0.039981	0.488477
TIMP1	blue	0.03984	0.488947
TNFAIP8L1	blue	0.007052	0.492375
PCDH18	blue	0.010637	0.492375
UNC5B	blue	0.012783	0.492375
NID2	blue	0.034216	0.492375
PDE5A	blue	0.041243	0.492375
